# Progressive ataxia of Charolais cattle highlights a role of KIF1C in sustainable myelination

**DOI:** 10.1371/journal.pgen.1007550

**Published:** 2018-08-01

**Authors:** Amandine Duchesne, Anne Vaiman, Magali Frah, Sandrine Floriot, Sabrina Legoueix-Rodriguez, Anne Desmazières, Sébastien Fritz, Christian Beauvallet, Olivier Albaric, Eric Venot, Maud Bertaud, Romain Saintilan, Raphaël Guatteo, Diane Esquerré, Julien Branchu, Anaïs Fleming, Alexis Brice, Frédéric Darios, Jean-Luc Vilotte, Giovanni Stevanin, Didier Boichard, Khalid Hamid El Hachimi

**Affiliations:** 1 GABI, INRA, AgroParisTech, Université Paris-Saclay, Jouy-en-Josas, France; 2 Sorbonne Université UMR S 1127, Paris, France; 3 Inserm, U1127, Paris, France; 4 CNRS, UMR 7225, Paris, France; 5 Institut du Cerveau et de la Moelle épinière, ICM, Paris, France; 6 TWB, Université de Toulouse, INRA, INSA, CNRS, Ramonville-Saint-Agne, France; 7 Allice, Paris, France; 8 LHA, Oniris, Université Nantes Angers Le Mans, Nantes, France; 9 BIOEPAR, INRA, Oniris, La Chantrerie, Nantes, France; 10 GenPhySE, Université de Toulouse, INRA, INPT, ENVT, Castanet Tolosan, France; 11 Centre de référence de Neurogénétique, Fédération de génétique, APHP, GHU Pitié-Salpêtrière, Paris, France; 12 EPHE, PSL Research University, Laboratoire de Neurogénétique, Paris, France; University of Liege, Faculty of Veterinary Medicine, BELGIUM

## Abstract

Hereditary spastic paraplegias (HSPs) are clinically and genetically heterogeneous human neurodegenerative diseases. Amongst the identified genetic causes, mutations in genes encoding motor proteins such as kinesins have been involved in various HSP clinical isoforms. Mutations in *KIF1C* are responsible for autosomal recessive spastic paraplegia type 58 (SPG58) and spastic ataxia 2 (SPAX2). Bovines also develop neurodegenerative diseases, some of them having a genetic aetiology. Bovine progressive ataxia was first described in the Charolais breed in the early 1970s in England and further cases in this breed were subsequently reported worldwide. We can now report that progressive ataxia of Charolais cattle results from a homozygous single nucleotide polymorphism in the coding region of the *KIF1C* gene. In this study, we show that the mutation at the heterozygous state is associated with a better score for muscular development, explaining its balancing selection for several decades, and the resulting high frequency (13%) of the allele in the French Charolais breed. We demonstrate that the *KIF1C* bovine mutation leads to a functional knock-out, therefore mimicking mutations in humans affected by SPG58/SPAX2. The functional consequences of *KIF1C* loss of function in cattle were also histologically reevaluated. We showed by an immunochemistry approach that demyelinating plaques were due to altered oligodendrocyte membrane protrusion, and we highlight an abnormal accumulation of actin in the core of demyelinating plaques, which is normally concentrated at the leading edge of oligodendrocytes during axon wrapping. We also observed that the lesions were associated with abnormal extension of paranodal sections. Moreover, this model highlights the role of KIF1C protein in preserving the structural integrity and function of myelin, since the clinical signs and lesions arise in young-adult Charolais cattle. Finally, this model provides useful information for SPG58/SPAX2 disease and other demyelinating lesions.

## Author summary

Hereditary spastic paraplegias (HSPs) are human neurodegenerative diseases mainly associated with lower extremity weakness and spasticity. Motor-sensory axons degeneration, implying heterogeneous cellular and molecular mechanisms and various genetic causes, is the neuropathological hallmark of this disease. Recently, mutations in *KIF1C* were associated with human spastic paraplegia type 58 (SPG58) and spastic ataxia 2 (SPAX2), where the radiological brain examination showed demyelination features. We report herein that progressive ataxia of Charolais cattle, a neurodegenerative disease with autosomal recessive inheritance, is caused by a substitution in the *KIF1C* gene, which leads to a functional knock-out. Interestingly this mutation is associated, in a heterozygous state, with a better muscular development, and thus a zootechnic advantage. Identification of the mutation will therefore be helpful to eradicate this disease. Further study of the lesions in ataxic bovine central nervous system highlighted a peculiar link to oligodendrocytes which were hypertrophied and harbored many membrane protrusions. The demyelinating plaques were enriched by these membranes and actin accumulation indicating close relationship between KIF1C, actin transport and axonal wrapping by oligodendrocyte tongues. Since *kif1c* knock-out mouse do not display any neurological symptoms, progressive ataxia of Charolais cattle thus provides a useful model for studying SPG58/SPAX2 and other demyelinating diseases.

## Introduction

Hereditary spastic paraplegias (HSPs) are clinically and genetically heterogeneous neurodegenerative diseases in humans. Clinically, the disease can be classified into pure or complicated forms. In the pure form of HSP, pyramidal tract signs are the clinical consequence of corticospinal axons degeneration. The complicated form of HSP is associated with neurological and extraneurological signs such as extrapyramidal manifestations, muscle weakness, sensory deficit, retinal degeneration, cognitive decline and cerebellar ataxia [1]. The pathological hallmark of HSP is thought to be the integrity breakdown of long axons of the upper motor neurons and of other brain structures in complex cases [2,3]. This failure is likely the result of the disruption of critical cellular mechanisms highlighted by functions of the identified causative genes and functional studies: vesicular trafficking and organelle shaping, axonal and cellular transport, lipid metabolism, mitochondrial function, brain development and myelination [4].

Mutations of genes encoding motor proteins, such as kinesin superfamily proteins (KIFs), have been implicated in various HSP clinical isoforms. *KIF5A* mutations account for autosomal dominant spastic paraplegia (SPG) 10 [5–7], mutations in *KIF1A* lead to autosomal recessive SPG30 or to autosomal dominant cases of complex HSP [8,9] and mutations in *KIF1C* have been found in autosomal recessive SPG58/SPAX2 [10–13]. Seven unrelated HSP families from the Middle East, Turkey, North Africa and Germany have been shown to segregate with either non-sense mutations, missense mutations or deletions in *KIF1C* gene. Most patients share cerebellar ataxia with pyramidal signs, and, when available, magnetic resonance imaging (MRI) examination reveals widespread T2 white matter hyperintensities, interpreted as demyelinating lesions [10]. Pyramidal tracts and cerebellar peduncles are the most affected brain regions and this may explain the association of spastic paraplegia and cerebellar ataxia in most patients. The precise histological changes underlying these white matter lesions remain undefined since no neuropathological report of human cases is available. In addition, the *kif1c* knock-out mouse [14] did not show any neurological or non-neurological manifestation, presumably because of the redundant expression of other kinesins in brain.

Livestock animals are also prone to develop hereditary diseases, and can provide good models for human diseases [15]. In bovines for example, due to repetitive linebreeding and the genetic bottlenecks in each breed, more than 500 traits or disorders have been described (OMIA http://omia.org). Amongst them, around 1/10 directly affect the nervous system. Causal mutations have been found for 15 of them, offering a parallel with comparable human diseases. For example, degenerative axonopathy in Tyrolean Grey cattle was found to result from a mutation in the *MFN2* gene [16], which is involved in Charcot-Marie-Tooth disease type 2 in humans [17]. Another form of axonopathy in the Rouge-des-Prés cattle is a consequence of a mutation in *SLC25A46* gene [18], reminiscent of mutations of the homologous genes in humans [19]. Bovine spinal dysmyelination, originally present in American Brown Swiss cattle, is due to a mutation in the *SPAST* gene [20], a mutation that causes SPG4 in humans [21].

However, the genetic basis for most of these hereditary diseases in livestock animals remains unclear, as was the case for progressive ataxia of Charolais cattle.

Progressive ataxia was first described in Charolais cattle in England in the early 1970s [22], and in the following years cases were reported in this breed worldwide [23–25]. Ataxia and paresis mostly involve the hind limbs. Limb incoordination progresses during the following few months, from early clinical signs, leading to permanent recumbency and ultimately to the animals having to be euthanized, as no effective treatment has been described [22,23,26].

Neuropathological changes are only microscopic and are confined to the central nervous system [23]. There is no peripheral nervous system alteration. Lesions consist of spread eosinophilic and acellular plaques about 20–80 μm in diameter, located within specific regions of the brain and the spinal cord, such as the cerebellar white matter and peduncles, the corpus callosum, the internal capsule and the ventral and lateral funiculi of the spinal cord. At the electron microscopy level, the lesions are composed of disordered myelin, hypertrophic and hyperplastic oligodendroglia tongues surrounded by many other cells, and are associated with nodes of Ranvier [23].

Progressive ataxia affects purebred cattle, or crossbred animals (at least three-quarters Charolais) and affects males and females equally [25]. Even if the genetic basis of this trait has been suspected for decades, its inheritance pattern was still unclear. A growing number of cases have been recorded by the French National Observatory of Genetic Diseases (ONAB www.onab.fr) since the early 2000s, in animals sired by artificial insemination or by natural service. Identification of the genetic basis of this disease was thus needed in order to eradicate the disease in the Charolais breed.

Here, we report that progressive ataxia of Charolais cattle results from a homozygous single nucleotide polymorphism in the coding region of the *KIF1C* gene, leading to a functional knock-out of this gene and therefore mimicking some of the mutations in humans. We took advantage of the existence of this natural model, firstly to determine the cytological process of the demyelination lesions associated with *KIF1C* mutation and, secondly, to provide an animal model to study spastic ataxia failure. Furthermore, our findings highlight the role of KIF1C protein in preserving the structural integrity and function of myelin and this model will provide useful information for other demyelinating diseases.

## Results

### Clinical summary

Seventy-one Charolais animals of both sexes presenting progressive ataxia symptoms as described in the literature: i.e. unsteady gait and stiff hind limbs, with a gradual worsening of the symptoms ultimately resulting in permanent decubitus, and irregular and “spastic” micturition in most of the females [22,23,26], were recorded over a period of 10 years by the French National Observatory of Genetic Diseases (S1 Table). The first symptoms appeared around age 18–24 months, but younger (as early as 6 months of age) as well as older cases (up to 5 years of age) were also observed. Evolution was highly variable, from a few weeks to more than 18 months. Diagnosis was confirmed by histopathological study for 10 cases for which nervous tissues were available. Pedigree analysis was undertaken using available data; unfortunately, some information was missing for cases bred by natural service or for some of the earliest recorded cases. This analysis was consistent with an autosomal recessive inheritance and involved a predominant founder ancestor, a bull born in 1964 (S1 Fig). This bull was a high contributor to the Charolais breed.

### A missense variant in the *KIF1C* gene is associated with progressive ataxia of Charolais cattle

Genotyping of 46 suspected cases was done using the Illumina bovine 50K SNP chip (including 8 cases confirmed by histopathological analysis) (S1 Table), followed by homozygosity mapping and haplotype analysis identified a single homozygous interval on bovine chromosome 19 (S2 Fig) shared by 41 animals. The minimal common interval was 681 kb long (chr19: 26848700–27529700), and contained more than 40 genes as predicted in the Ensembl database. Whole-genome sequencing was performed on two affected animals (aged 31 and 36 months), and one wild type (WT) animal. Sequence analysis focused on this interval on chromosome 19. The detected polymorphisms (SNP and small indels) were then filtered in several steps. First, affected cattle had to be homozygous for the causative variant, while the phenotypically WT cattle had to bear at least one WT allele. Second, since this mutation is supposedly specific to the Charolais breed, polymorphisms were discarded if they were already present in the dbSNP database and/or in the Illumina SNP chip. Finally, polymorphisms were filtered according to their predicted effects on transcript and/or protein, based on the hypothesis that this mutation is deleterious (Table 1).

**Table 1:** Variants detected by whole-genome sequencing of 3 animals, including 2 with progressive ataxia.

Filtering steps	No. polymorphisms
**Polymorphisms in the 681kb homozygous interval**	1503
**Polymorphisms homozygous in both affected cases, and absent or heterozygous in WT animal**	367
**Polymorphisms absent from dbSNP database**	159
**Polymorphisms different from those of Illumina SNP chip in breeds other than Charolais**	146
**Non-synonymous coding polymorphisms**	1

**Fig 1:**
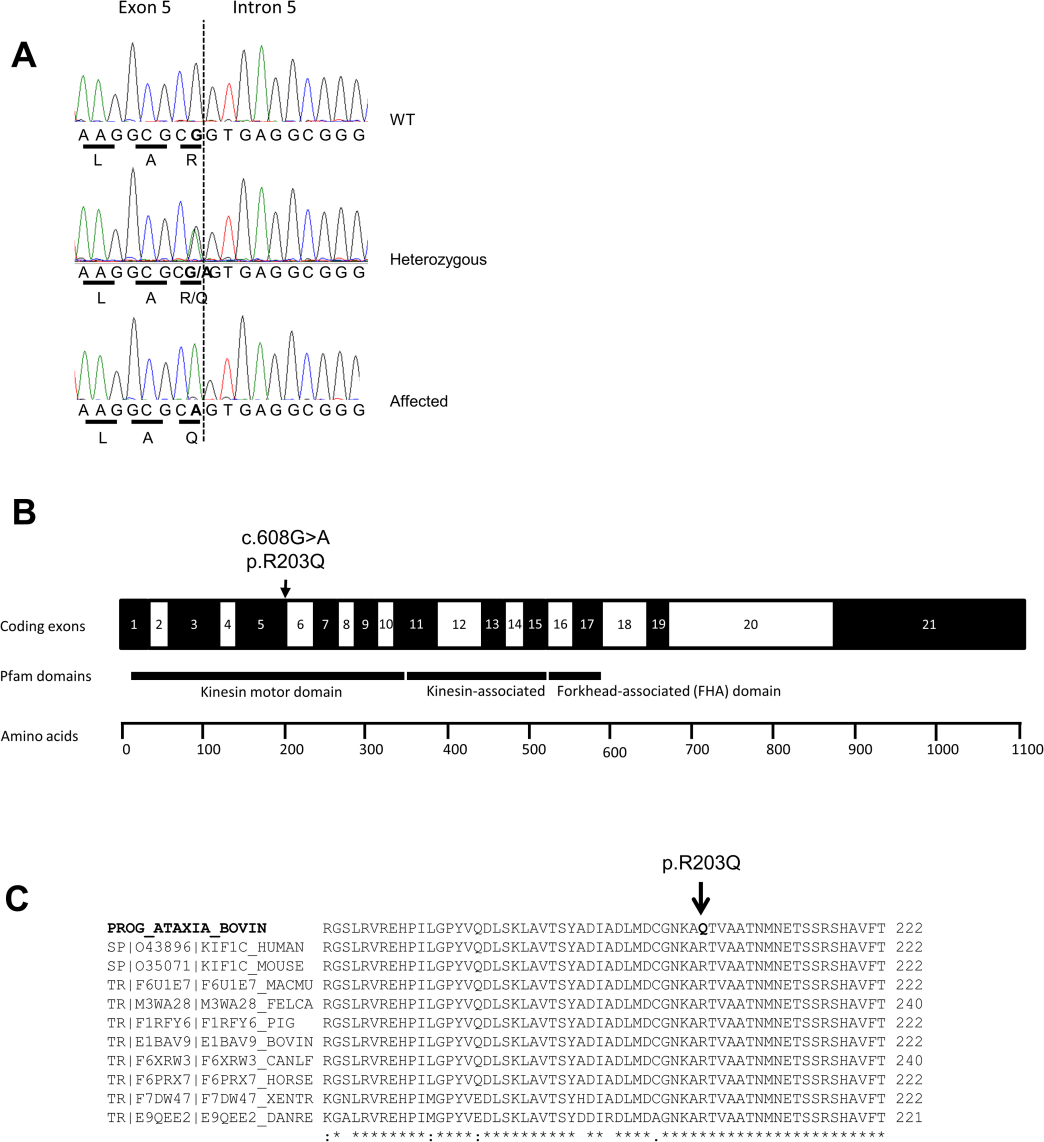
Identification of a variant in *KIF1C* gene in bovine animals affected by progressive ataxia. (A) Sanger sequence electropherogram traces for the causal mutation in the bovine *KIF1C* gene done on a wild type (WT), a heterozygous carrier and an affected animal. The G>A substitution affects the last nucleotide of bovine exon 5. Translated amino acids are presented below the genomic sequence. (B) Schematic diagram of coding exons from *KIF1C* gene in cattle (protein with 1104 amino acids) with the predicted functional domains of the protein, with the position of the mutation indicated (*arrow*). (C) Based on protein alignment, the affected amino acid is highly conserved in vertebrates and located in a conserved region of the protein. MACMU, *Macaca mulatta*; FELCA, *Felis catus*; BOVIN, bovine (*Bos taurus*); CANLF, *Canis lupus familiaris*; XENTR, *Xenopus tropicalis*; DANRE, *Danio rerio*.

The only remaining putative causal SNP was a single substitution in exon 5 of *KIF1C* (chr19:27041449 C/T). For easier comprehension and since *KIF1C* gene in cattle is on the reverse strand, the substitution will be referred as *KIF1C* G>A in order to match with the transcription sense (Fig 1A). The G>A substitution was further tested (Sanger sequencing) on 143 Charolais animals, including 70 of the 71 cases suspected of progressive ataxia for which DNA was available. All 41 genotyped cases bearing the 681-kb homozygous interval and 19 non-genotyped cases were homozygous for the G>A variant, and all their parents were heterozygous. On the contrary, the 5 genotyped cases that did not bear the homozygous chromosome 19 haplotype and 5 non-genotyped cases were either heterozygous or homozygous for the WT allele. These cases were either very young (#32, #59, #60) or old (#22, #33, #34) with a very slow progression of the disease (#67) which differs from the “classical” ataxic phenotype, suggesting different genetic origin (S1 Table).

In conclusion, most of the suspected ataxia cases (60/70) were homozygous for the *KIF1C* G>A substitution, including the histopathologically confirmed cases.

The other 10 cases were either heterozygous for this mutation or WT, which indicates the possibility of other neurodegenerative syndromes in the Charolais breed, especially because the analysis of the genotypes in these cases was not concordant with the defined genetic interval.

The polymorphism was then inserted in a bovine custom SNP chip, and tested on animals from 7 French breeds, including the Charolais breed. The frequency of the *KIF1C* mutation was estimated to be around 13% in the Charolais breed. The mutation was not found in other breeds, except 3 heterozygous cases in the Blonde d’Aquitaine breed, which has Charolais blood in its ancestry (Table 2).

**Table 2:** Genotyping of animals of 7 French breeds for the KIF1C mutation.

Breed	No. homozygous mutated cases	No. heterozygous cases	No. homozygous WT cases
**Charolais**	45	854	2698
**Blonde d’Aquitaine**	0	3	2436
**Limousin**	0	0	303
**Brown Swiss**	0	0	946
**Montbéliarde**	0	0	47046
**Normande**	0	0	12891
**Holstein**	0	0	61136

### The missense variant in the *KIF1C* gene is associated with growth and morphology traits in the Charolais breed

Taking account of the relatively high frequency of the *KIF1C* substitution in the Charolais breed, we then sought to determine whether it was associated with production traits in this breed. In a sample of 3300 Charolais animals from the French commercial population, the number of homozygous mutated individuals represented about 1.1% of the samples for young animals at 7 months and slightly below 1% at 24 months, whereas no homozygous case was observed at 30 months of age. Accordingly, the mutated allele frequency was around 13% up to 24 months of age and decreased to 12% at 30 months (Table 3).

**Table 3:** Association between the ataxia variant and morphology traits in animals of the Charolais breed.

Trait	Mean	Phenotypic std	n(GG)	n(AG)	n(AA)	freq(A)	AG-GG	AG-GG / mean	AG-GG / std
**Birth weight (kg)**	48	3.8	2540	782	37	12.7	0.04	0.1%	0.01
**Muscular development score at 7 months**	63	9.4	2309	722	34	12.9	1.6**	2.5%	0.17
**Skeletal development score at 7 months**	69	8.9	2309	722	34	12.9	-0.26	-0.4%	-0.03
**Weaning weight at 7 months (kg)**	315	24.9	2206	698	32	13	1.6*	0.5%	0.06
**Weight at 18 months (kg)**	600	39.5	331	112	6	13.8	2.67	0.4%	0.07
**Weight at 24 months (kg)**	618	45.2	173	62	2	13.9	12.5*	2.0%	0.28
**Muscular development score at 30 months**	60	7.1	189	60	0	12	1.31	2.2%	0.18
**Skeletal development score at 30 months**	69	7.9	189	60	0	12	1.78*	2.6%	0.23

Phenotypic std stands for Phenotypic Standard Deviation; n(GG) stands for the number of animals homozygous for the WT (G) allele; n(AA) for the number of animals homozygous for the ataxia (A) allele; n(AG) for the number of heterozygous animals. Freq(A): frequency of the ataxia (A) allele; AG-GG: contrast between heterozygous (AG) and WT (GG) genotypes; AA-GG: contrast between ataxia (AA) and WT (GG) genotypes; AG-GG mean and AG-GG std: effect of the ataxia allele (at the heterozygous state) respectively on the phenotypic mean and standard deviation. Significant contrasts between heterozygous and WT genotype are presented in bold. * p<0.05 ** p<0.01

The contrast between heterozygous and WT homozygous genotypes was significant for muscular development score at 7 months (p<0.01), weaning weight at 7 months (p<0.05), weight at 24 months (p<0.05) and skeletal development score at 30 months (p<0.05). The effects of the mutant allele represented 2 to 2.6% of the mean and 17–28% of the phenotypic standard deviation. Thus, the high frequency of the allele responsible for ataxia may be at least partially attributed to selection on beef traits. Due to smaller numbers of individuals, none of the contrasts between homozygous genotypes (WT and ataxia) was significant (S2 Table).

### The *KIF1C* mutation results in loss of function

The *KIF1C* gene, which is relatively conserved in mammals, encodes a microtubule-based motor protein belonging to the kinesin family [27] (respectively 1103 and 1105 amino acids long in human and bovine). This protein harbours an N-terminal kinesin motor domain, a central kinesin associated domain and a forkhead-associated (FHA) domain [14]. The G>A *KIF1C* substitution leads to the replacement of an arginine by a glutamine (p.Arg203Gln), within the kinesin motor domain (Fig 1B). This amino acid is highly conserved across evolution (Fig 1C). Based on PROVEAN prediction software [28], this substitution is expected to be deleterious and affects the function of KIF1C (score = -3.822). Moreover, the substitution affects the last nucleotide of exon 5, thus exon splicing is predicted to be impaired, inducing a reduction in the predicted splice score, which dropped from 7.8 to 4.8 when analysed with Splice Site Score Software (http://rulai.cshl.edu). The result of analysis with ESE-finder software [29] suggests that the mutation may also modify exonic splicing enhancer (ESE) motifs at the junction between exons 5 and 6, which would alter the affinity with several serine/arginine-rich (SR) proteins (S3 Fig).

**Fig 2:**
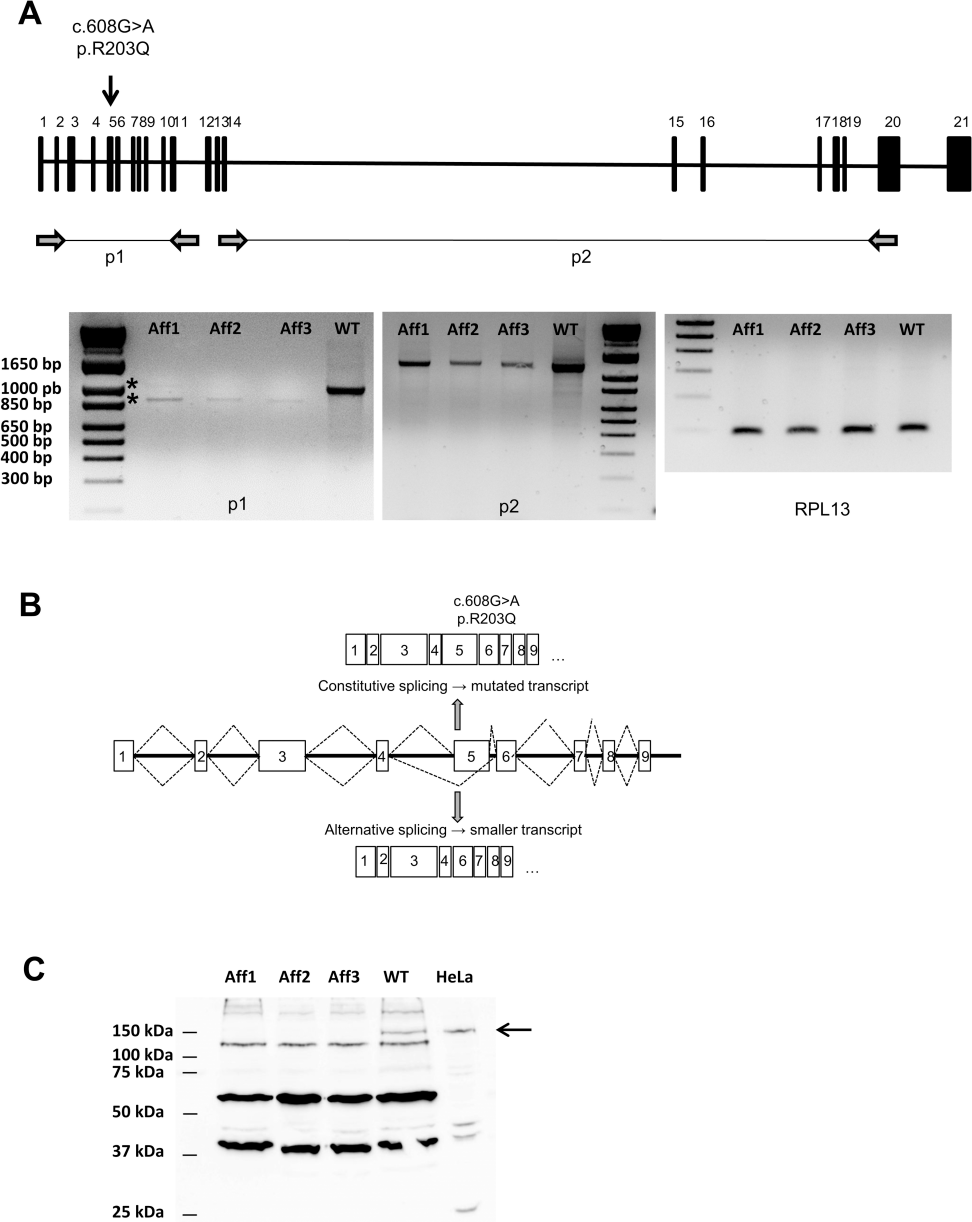
*KIF1C* variant affects mRNA expression and leads to a functional knock-out. (A) Schematic diagram of the *KIF1C* gene in bovine sequence, located on chromosome 19, with the mutation indicated by an arrow. Primer pairs p1 and p2 (respectively amplifying *KIF1C* exons 1 to 11 and exons 13 to 20) are shown downstream of the diagram. RT-PCR from WT and affected bovine brains with p1 and p2 primer pairs demonstrated that *KIF1C* expression is modified in affected animals both in quantity–with mRNA decay–and quality (several transcripts in affected animals). *RPL13* (ribosomal protein L13) was used as a housekeeping gene. (B) Schematic diagram of *KIF1C* transcripts in affected bovine. The normal transcript bears the G>A mutation and leads to a mutated protein; the alternative transcript results from defective splicing and leads to exon 5 skipping. (C) Proteins were extracted from brains of WT and affected bovines, and from HeLa cells. Samples were analysed by immunoblotting with antibody against KIF1C proteins. No KIF1C protein was found in affected animals. WT, wild type; Aff, affected.

*KIF1C* is ubiquitously expressed in human and in mouse [14,27]. *KIF1C* mRNA expression was studied in affected and WT bovine brains, as this organ, together with lymph nodes, is known to present the main histological alterations in our model [23]. Using primers spanning the mutation (i.e. amplifying exons 1 to 11), we showed that contrary to WT animals, affected ones could display two transcripts: a hardly detectable full-length transcript, visible in one affected case (same size as expected), and a shorter transcript (Fig 2A). After sequencing, the full-length transcript was shown to bear the G>A substitution. The shorter transcript resulted from abnormal splicing and its exon 5 was skipped (Fig 2B), leading to a premature stop codon at position 204 (p.RT203-204QStop). Transcripts were less abundant in affected cases than in WT, probably also resulting from mRNA decay. The low mRNA quantity was confirmed with the amplification of exons 13 to 20, downstream of the mutation, with less abundant transcripts (<50%) in affected animals than in WT.

KIF1C protein expression was then assessed in brain extracts from WT and affected animals, as well as in HeLa cell extracts (Fig 2C). As expected based on the mRNA expression level, KIF1C protein was undetectable in affected animals, either at the expected size as in WT animals and HeLa cell extracts (145 kDa) or at a lower size, as the transcript resulting from exon 5 skipping encodes a 151 amino acids truncated protein (S4 Fig).

### Neuropathological studies

Briefly, the classical histopathological findings made on frozen or paraffin-embedded brain sections of cases aged from 9 months to 35 months were concordant with the description of Blakemore *et al*. [23], with the presence in white matter of diffuse patches of myelin pallor in all studied regions (cerebellum, cervical spinal cord, corpus callosum and internal capsule). These demyelinating plaques, measured 20 to 80 μm in diameter and often coalesced (asterisks and lined up asterisks) (S5 Fig).

**Fig 3:**
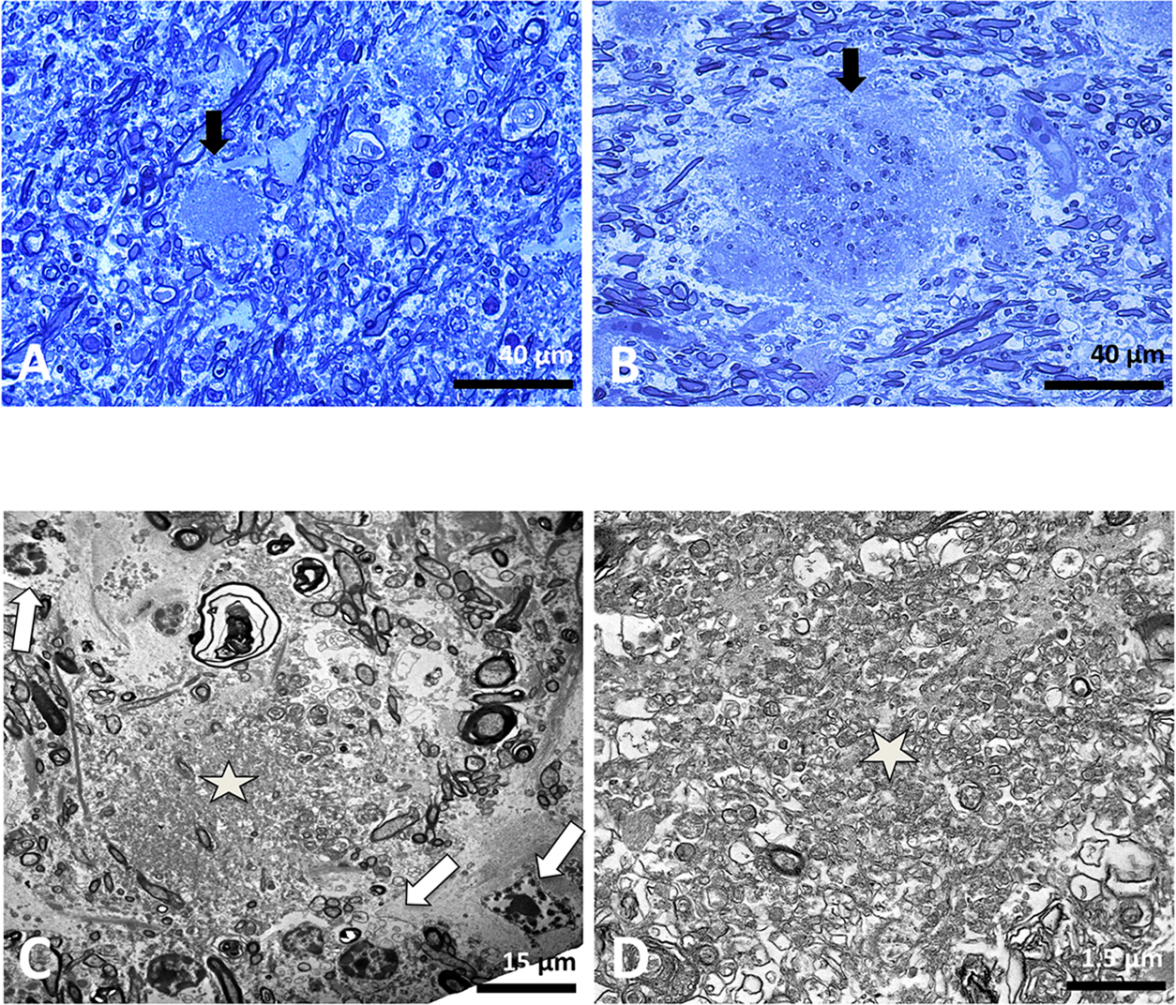
Cytological features of the lesions. Semi-thin blue toluidine stained section of white matter from spinal cord and cerebellum. (A) Cerebellar white matter less affected region showing a small lesion or “pre-plaque” (*arrow*) as a hypertrophied cell with histological characteristics reminiscent of an oligodendrocyte. (B) Spinal cord white matter showed a “mature” lesion constituted by amorphous and acellular material engulfing many myelin fibres and cellular debris (*arrow*). The surrounding nerve fibres were more or less disturbed. Electron micrographs of oligodendrocyte modifications consisting of intracytoplasmic inclusions. (C) Electron micrographs of frontal section of mature demyelinating plaque. The major part of the centre of the lesion (*star*) is composed of membranous, vesicular structures and fibrillary elements, but no cytological organelles (e.g. mitochondria or endoplasmic reticulum) were identifiable and no membranous binding was observed. Immediately around the centre, there are many myelinic and amyelinic processes, some of which are degenerated. The last “ring” is composed of many surrounding cells (*white arrows*) and complete the white matter lesion. Some cells are easily recognizable as astrocytes, their cytoplasm containing gliofilament tangles; some others could be histologically reminiscent of oligodendrocyte cells while others are not recognizable in the absence of specific markers. (D) High magnification of the area marked by a star in (C) evidenced myelinic bodies, small vesicles intermingled with membranous processes and fibrillary and amorphous material. These ultrastructural features of lesions were similar irrespective of the studied brain region (cerebellum, spinal cord and internal capsules). Scale bar: (A) and (B) 40 μm, (C) 15 μm, (D) 1.5 μm.

On samples embedded for electron microscopy, at semi-thin or ultrathin sections and according to their size and heterogeneity, we could distinguish like Blakemore *et al*. [23], two types of plaques: small ones or “pre-plaques” (Fig 3A), and complex ones or “mature” plaques (Fig 3B). Small plaques are mainly composed of hypertrophied oligodendrocytes, with tongues protrusion, surrounding thin myelinated axons (Fig 3C). Mature plaques have a diameter that could reach 80 μm (Fig 3D). Their centre is occupied by vesicles and membranous structures associated with cellular debris.

**Fig 4:**
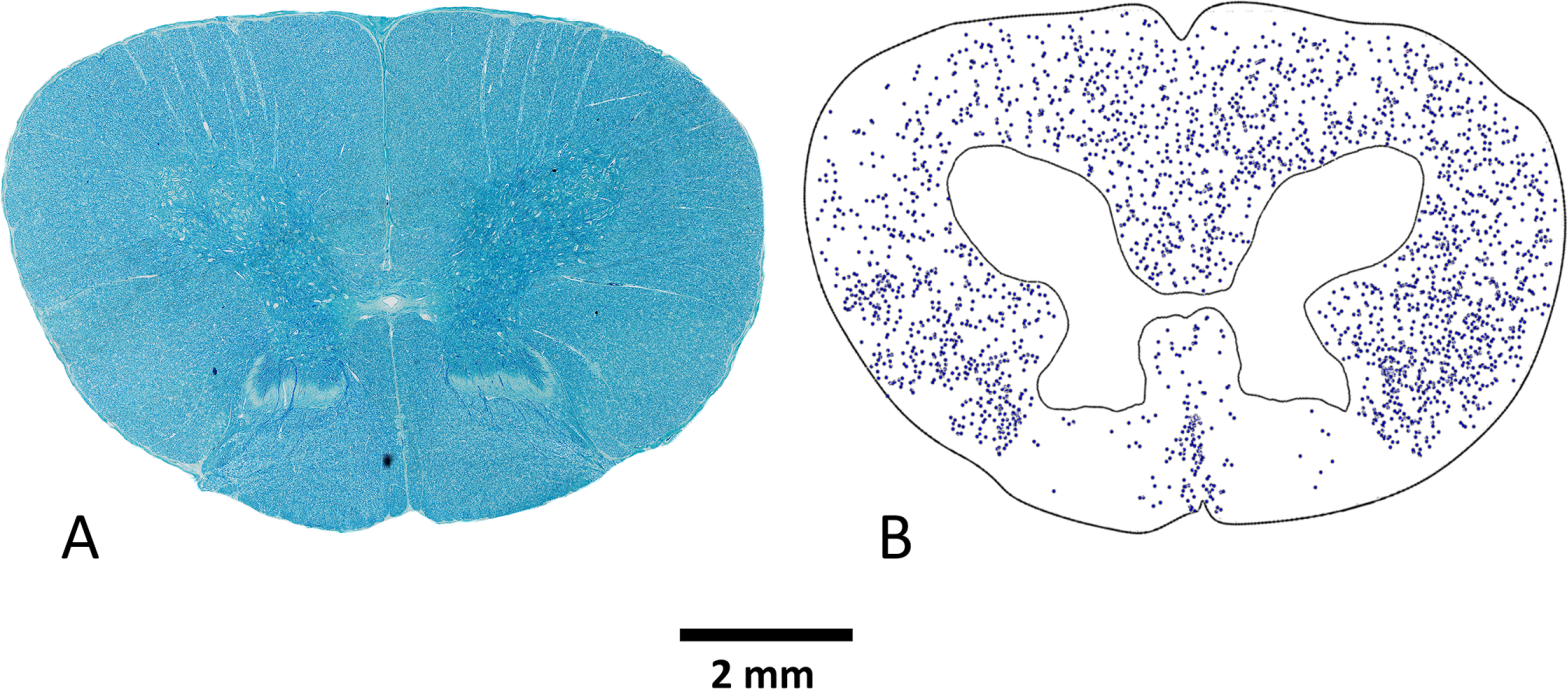
Topographical distribution of lesions in spinal cord. (A) Cervical spinal cord on paraffin-embedded section and Kluver-Barrera staining. Myelin pallor was diffuse and many tracts, presumably ascending and descending ones, were affected by demyelinating plaques. White matter stacks of various sizes were sparsely distributed. (B) Drawing of white matter lesions one by one, as presented in S5C Fig (*dark arrows*), showed that the ventral and lateral tracts harboured the majority of lesions. The dorsal tracts, except the median part, were less affected, but nevertheless contain very few stacks. Scale bar: (A) and (B) 2 mm.

We have drawn point by point the topographic distribution of demyelinating lesions on Kluver-Barrera-stained frontal sections of the cervical spinal cord of one animal. Except for the medial longitudinal fasciculus and the tectospinal tract, all funiculi (sensory and motor) were affected (Fig 4). This medullar white matter lesion distribution was more widespread than the canonical myelin pallor sign reported in human hereditary spastic paraplegia cases, where the lesions are generally limited to lateral and ventral tracts [30], and also differed from those observed in amyotrophic lateral sclerosis (ALS), where the preservation of posterior columns and the spinocerebellar tracts is usual [31].

In spite of the severity of the myelinic lesions, the grey matter was minimally affected. Rare neurons showed mild vacuolisations but neuronal loss was limited. Semi-quantitative analysis of Purkinje cell loss in cerebellum was unremarkable except in some old animals, where this cell loss could be related to aging. There were no neuropathological stigmata in neurons, such as intranuclear or intracytoplasmic inclusions.

### Cellular and subcellular component of the lesions

The core of lesions did not contain cellular nuclei, but was often surrounded by cells (Fig 3C). At the EM level, the cytological characteristics of some of these cells suggested they were astrocytes, microglia or oligodendrocytes. To confirm the nature of the surrounding cells, we used specific cellular markers. First, markers against lymphocytes T (CD3 marker) did not reveal their presence in the vicinity of lesions or in other brain regions.

**Fig 5:**
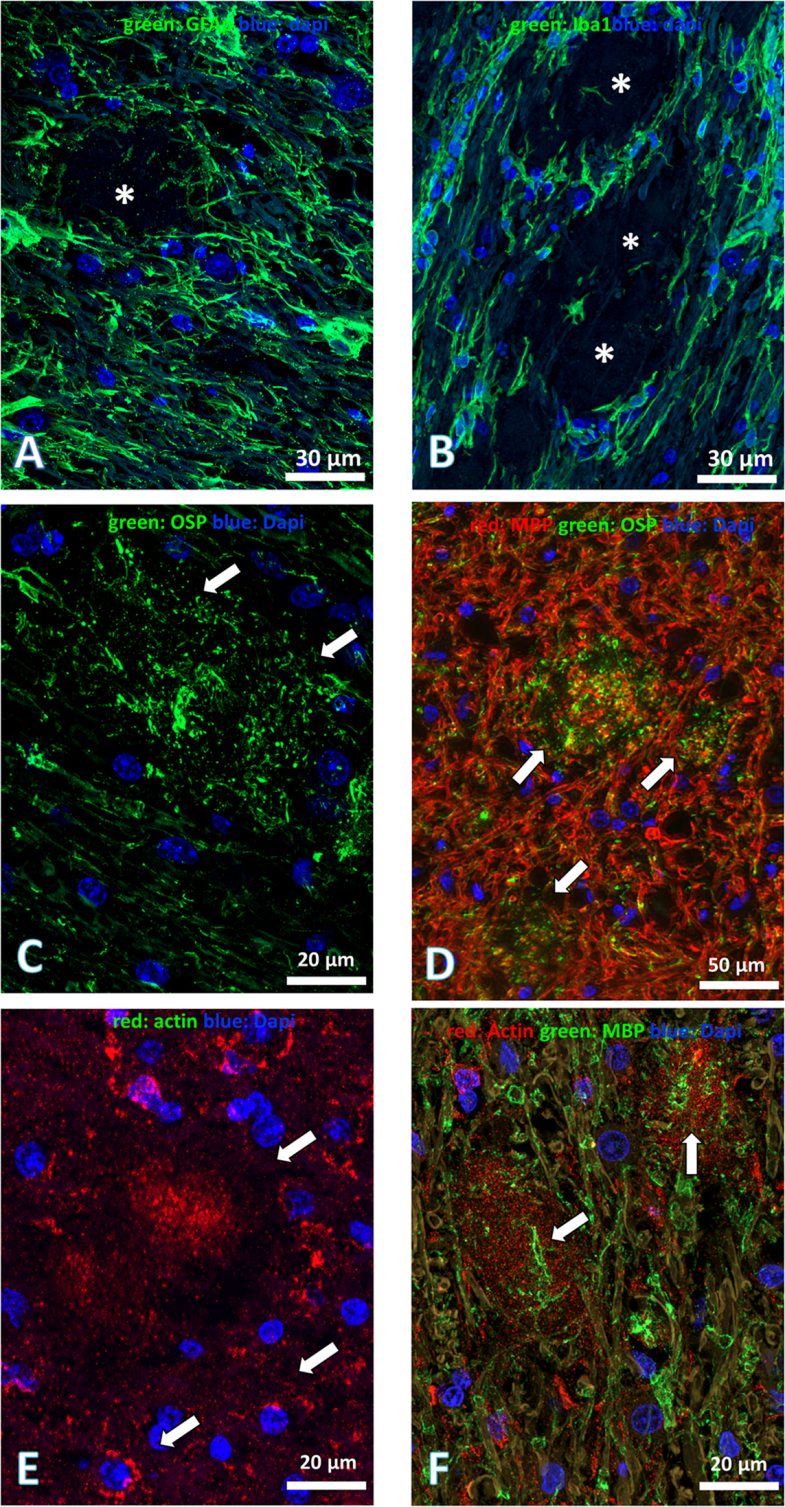
The participating cells in lesions. (A) Prominent astrocytic reaction as evidenced by anti-GFAP antibody (GFAP, green; Dapi, blue). Astrocytic reaction, engulfing the lesion; a few astrocytic feet penetrated the lesion (*white asterisk*). (B) Microglial activation, immunolabelled by anti Iba1, reproduced similar topography as the astrocytic reaction by surrounding the lesion, and few microglial cell processes infiltrated the lesion (*white asterisk*) (Iba1, green; Dapi, blue). Concurrently, astrocytic and microglial cell activations are present not only around the lesions but also elsewhere. (C) Immunostaining of anti-oligodendrocyte specific protein (anti-OSP), a cell membrane oligodendrocyte marker (OSP, green; Dapi, blue. Big or confluent lesions (*white arrows*), where the centre is occupied by many OSP-positive and intricate processes of different shapes and sizes, which presumably depend on the plane section. (D) Double immunostaining showed that the two markers–cytoplasmic (MBP) (red) and membrane (OSP) (green)–of oligodendrocytes were present together in the demyelinating lesions and the centre of the plaque was double-immunostained (*white arrows*). (E) Anti-actin immunostaining (red), Dapi (blue), actin protein aggregates were also accumulated in the centre (*arrow*) of the plaque and in the cytoplasm of some surrounding cells (*white arrows*). (F) Double immunostaining of actin (red) and MBP (green) Dapi (blue). Within the core of the plaque with actin immunostaining, note the presence of oligodendrocyte processes (*white arrows*). Scale bar: (A) and (B) 30 μm, (C) 20 μm, (D) 50 μm, (E) and (F) 20 μm.

In affected animals, anti-GFAP antibody showed diffuse and more intense labelling in white matter compared to grey matter, indicative of an astrocytic response (Fig 5A). The astrocytic feet encircled the lesion but rarely penetrated the core of the mature plaque. Microgliosis as revealed by Iba1 was also present and its intensity was similar to astrocytosis labelling (Fig 5B). Following the astrocyte feet location, the microglial processes surrounded the lesion, but they never penetrated the core of the plaque.

The oligodendrocyte-specific protein (OSP) antibody, marker of oligodendroglial cells membrane, gave relevant immunostaining (Fig 5C). Plasma membrane of oligodendrocytes as well as the core of the plaques were labelled, indicating that the membranous structures of the core of plaque, observed at the ultrastructural analysis, was mainly formed by oligodendrocyte cell membranes. The anti MBP (myelin basic protein) marker of myelinic membrane, also showed no normal myelinic fibre staining in plaques. However, much cellular MBP-positive debris was observed thus confirming the demyelinating nature of these plaques. Double immunostaining clearly revealed the presence of the two antigens (MBP [red], OSP [green]) in the plaques (Fig 5D).

Since the core of plaques is also composed of vesicles and fibrillary and amorphous material, we used antibodies directed against cytoskeleton proteins (neurofilament, microtubule, actin), but only antibody directed against actin, crossed with bovine protein and evidenced aggregates in cells surrounding the core of plaques. Some of the cells, according to their localization and the size of their nucleus, could be tentatively identified as oligodendrocytes. More interestingly, the centre of plaques was also uniformly immunostained. The immunostaining was generally vesicular in shape and thinner in the core of plaques compared to that in cells (Fig 5E and 5F).

### Abnormal extension of paranodal sections

**Fig 6:**
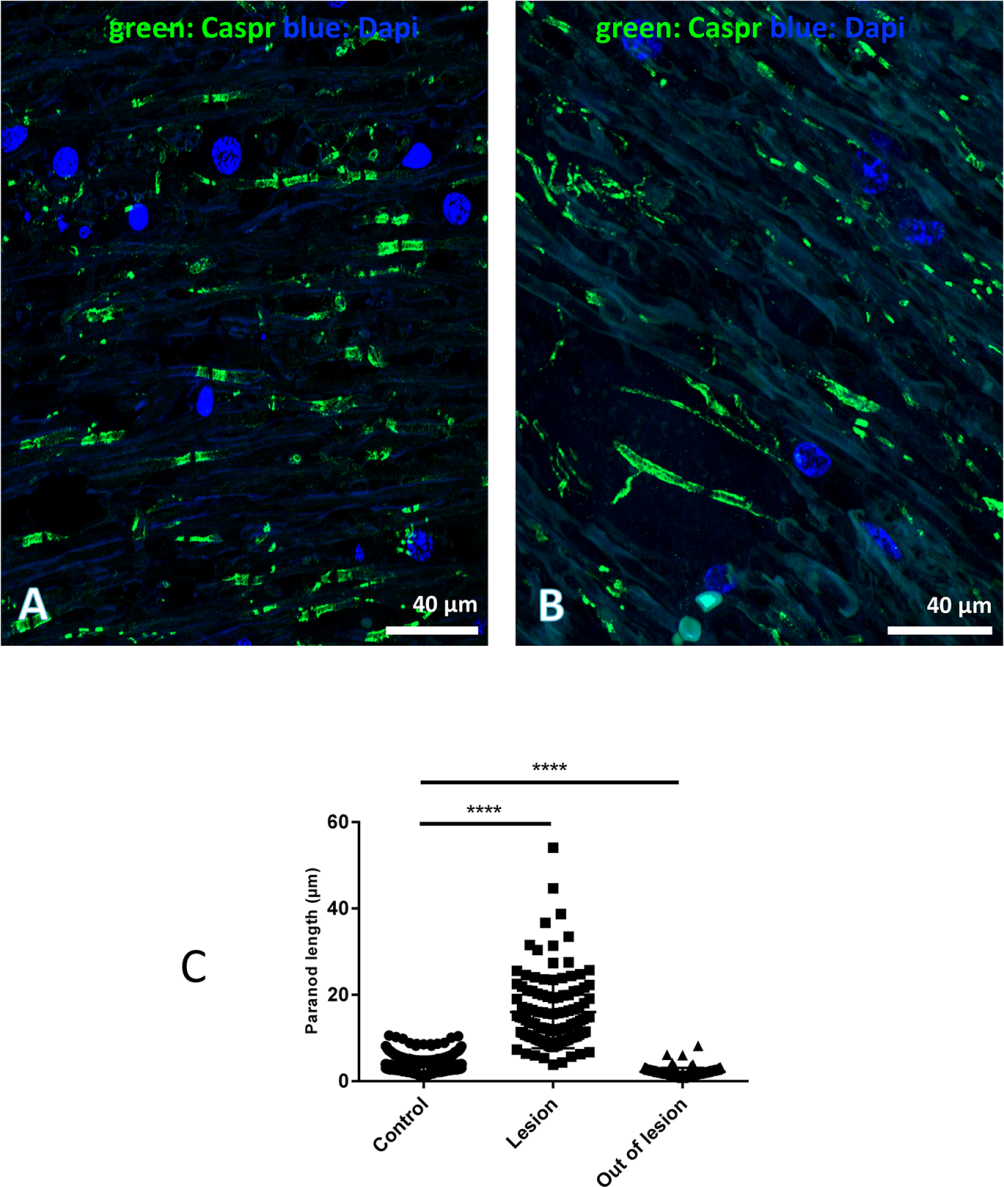
Quantification of the length of paranodal section in cerebellar peduncles of non-affected and affected cattle. (A) Caspr positivity was concentrated in two paranodal compartments on either side of the node of Ranvier. The immunostaining was observed on myelinated fibres of various diameters. (Caspr green, Dapi blue). (B) The lengths of the paranodal region vary slightly; however, in affected white matter this length appeared more variable and greater within and around the demyelinating lesion. (C) Quantitative comparison and graphic representation of these lengths in WT controls, within and outside the lesions. Numbers of quantified paranodal sections in WT controls: 570; in affected cattle: inside lesion 115 paranodes and outside lesion 194 paranodes. Scale bar: (A) and (B) 40 μm.

Since oligodendroglial changes were also accompanied with an increased length of the paranodal section and nodes of Ranvier and accumulations of osmiophilic and membranous structures facing the node of Ranvier [23], we characterized these structural modifications by using anti Caspr/paranodin as a marker of the paranodal region (Fig 6A and 6B). We showed that the modifications at the node of Ranvier were not myelin fibre thickness dependent (Fig 6B). We then measured and compared only the length of the paranodal region and not the thickness of myelin fibres. Most of the nodes of Ranvier had normal structures, reminiscent of those in the brain of non-affected animals. However, in the plaque or in its vicinity, the length of paranodal immunostaining was greater (16+/-0.8 μm, range 8.3–38.7 μm n = 570 nodes) and up to four times the length in WT animals (3.9+/-0.6μm, range 1.5–6 μm, n = 155 nodes) or outside the plaques in affected animals (2.1+/-0.7 μm, range 0.6–6.1 μm, n = 194) (Fig 6C).

## Discussion

### *KIF1C* loss of function accounts for progressive ataxia in Charolais cattle

We gathered a series of cases of suspected progressive ataxia in animals of the Charolais breed.

Using a combination of next generation sequencing and whole genome SNP analysis, we determined that in the majority of cases with this phenotype, the disease is caused by a single substitution, c.608G>A, in the *KIF1C* gene. This substitution has two effects: firstly, it modifies a conserved amino acid (p.R203Q). Secondly, it causes an alternative splicing event, resulting in exon 5 skipping in most of the transcripts (p.RT203-204QStop) associated with a drastic reduction of overall mRNA expression and leading to the absence of detectable KIF1C protein in brain extracts from affected cattle.

*KIF1C* human gene mutations, including nonsense, missense, splicing or frame shift types, were mainly localized in the kinesin motor domain [11,13,32], but coiled-coil, PTDP1 and FHA domains were also affected by deletion or missense mutations [11–13] (S6 Fig). The majority of the known mutations, localized in the microtubule-based motor domain disturb adenosine triphosphate hydrolysis of the microtubule. It was clearly demonstrated that the Arg301Gly mutation [32], for example, reduce microtubule binding affinity of KIF1C, and transports rates consequently. The mutation in Charolais cattle, also localized in the motor domain. It is leading, however, to the total absence of protein and contrary to KO mice [14], this absence was pathogenic.

Progressive ataxia in cattle was described in the 1970s in the Charolais breed, and the mutation now segregates with a high frequency in the present Charolais population (13%). Association studies in the studied cohort showed that this mutation is positively correlated with better scores in muscular development at weaning (p<0.01), weaning weight at 7 months (p<0.05), weight at 24 months (p< 0.05) and skeletal development at 30 months (p<0.05). These production traits have been positively selected in Charolais cattle for several decades and it is then tempting to postulate that the *KIF1C* mutation may have been selected with a hitch-hiking effect concomitantly with a close beneficial mutation for the Charolais breed [33].

Alternatively, it may play a direct role in muscular development and lead to a positive effect–at least at the heterozygous state–on muscular growth. Charolais cattle, like other beef cattle, are selected for their potential for muscular growth, and in theory this selection could affect muscle characteristics themselves [34]. This hypothesis was strengthened by a recent study showing that KIF1C, alongside other microtubule motors, is involved in muscle differentiation [35]. The effect of the *KIF1C* mutation in bovine muscle has yet to be assessed but *kif1c* knocking down by siRNA in C2C12 rodent muscle cells affects nucleus dynamics and number [35]. Interestingly, other data show a higher expression level of KIF1C in bovine muscles compared to the other tissues (InnateDB; www.innatedb.com). Our findings demonstrate that *KIF1C* mutation lead to the absence of protein, thus the heterozygous state should conduct to the lower expression levels compared to the homozygous state. Such knock-down has been associated with higher muscle development for other genes preferentially expressed in this tissue, namely *GDF8* gene, encoding Myostatin protein [36].

Interestingly, genetically different ‘progressive ataxia-like diseases’ are expected to segregate in the Charolais breed as 10/70 of the cases were not explained by mutations in *KIF1C*. We cannot exclude the possibility that deep intronic mutations may be involved in these negative cases or that mutations in other genes are also responsible for this kind of phenotype.

### Bovine progressive ataxia is the first animal model for SPG58/SPAX2

The first clinical signs observed in *KIF1C* mutated bovine were often an ataxic gait associated with weakness of the hind limbs, reminiscent of spasticity, which progressed slowly to the typical phenotype of spastic ataxia and ultimately led to recumbency. In humans, *KIF1C* mutations account for spastic ataxia SPAX2 or complex hereditary spastic paraplegia SPG58 [10–13]. The phenotype in patients is heterogeneous, associating spasticity and in some instance ataxia, thus resembling to the phenotype observed in cattle [10].

SPG58/SPAX2 patients present signs of widespread demyelination on neurophysiological examination and brain MRI (T2 hyperintensities), sometimes accompanied by a mild cerebellar atrophy. MRI signs of demyelination involved many tracts (e.g. corpus callosum, internal capsule, occipital white matter, cerebellum and the brainstem), as it is generally the case for HSPs, even if the corticospinal tract degeneration appears to be pivotal [30,37–39]. However, up to now, no histopathological study has been reported for SPG58/SPAX2.

The histopathological study was performed in bovine spinal cord to precise the anatomical counterpart for spasticity. In affected cattle, the topographic distribution of white matter lesions in spinal cord is not restricted to the corticospinal tracts as it is the case for human spastic paraplegia. Indeed, other descending and even ascending tracts, except the medial part of ventral white matter, are affected. This could be due to the anatomical differences between the spinal cords of human and quadruped animals where the corticospinal tracts are not fully lateralized [40]. Even so, this peculiar topographic distribution could explain the spasticity in cattle.

Contrarily, *kif1c* knock-out mice do not present any neurological symptom up to 18 months old [14]. This unexpected discrepancy could be explained by a functional redundancy with other proteins in this species.

Thus, ataxic bovine can be considered as the first and natural animal model for human SPG58/SPAX2.

### Bovine progressive ataxia as a peculiar demyelinating disorder

The rare publications of HSP human cases have mainly dealt with the characterisation of neuronal death and proposed an axonal dying back mechanism as the pathological explanation [30]. However, other mechanisms participating in the neuronal death may exist and demyelination might be one of them as it has been involved in HSP demyelinating or defective myelination entities such as SPG1 [41] and SPG2/Pelizaeus-Merzbacher disease [42].

Our study provides further details of the unusual nature of the lesions in ataxic bovines: primary oligodendrocyte hypertrophy and tongue protrusion result in demyelination and the disease could tentatively be considered as a cell autonomous oligodendroglial disease. Hypertrophy of oligodendrocytes precedes demyelination in several conditions, such as the viral demyelinating disease progressive multifocal encephalopathy, where the nucleus and cytoplasm hypertrophy of oligodendrocytes is due to JC virus infection [43]. Compared to other demyelinating diseases, the ataxic lesions are also smaller in size and more diffuse, with a predominance in the spinal cord, internal capsule and cerebellum. Moreover, no associated inflammatory process was observed in ataxic bovines, which is unusual as it is central to the pathological process in other demyelinating disorders such as MS [44]. In addition, the demyelination process does not result from abnormal brain development, since the majority of affected animals developed the first clinical signs when aged around 18 months, with some rare cases at around 8 months, largely after the myelinating process had finished in this species [45]. Consequently, demyelinating lesions in ataxic bovine differ largely from any other known demyelinating disease.

### Nodal and paranodal sections involvment

Both myelin sheath attachment to axon and saltatory impulse conduction depend on nodal, paranodal and juxtaparanodal protein expression and proper subcellular targeting. The axonal glycoprotein contactin-associated protein (Caspr/paranodin) was used as a marker to explore the paranodal junction. Caspr is a protein highly enriched in paranodal sections of myelinated axons in central and peripheral nervous system, more particularly located in a region where the paranodal myelin loops adhere to the axonal plasma membrane and form a physical barrier between the nodal Na+ (Na_v_) channels and the juxtaparanodal K+ (K_v_) channels. We found a significant elongation of Caspr/paranodin-positive stretches in demyelinating plaques. Naked nerve fibres with stretches of Caspr were often found in the plaque and its vicinity, suggesting that Caspr persists for some time after demyelination. The increase in Caspr/paranodin-positive region length has variously been interpreted as evidence of remyelination in experimental animal models of demyelination/remyelination following nodal Na_v_ channel protein and then after K_v_ channel protein accumulation [46,47]. On the other hand, diffuse nodal and paranodal proteins Na_v_, K_v_ channel, Caspr/paranodin and Caspr2 were reported to be distributed along denuded axons and this was considered to be an early event of the demyelinating plaques in MS [48]. The absence of specific antibodies directed against juxtaparanodal Na_v_ and K_v_ channel proteins in cattle brain prevents us from exploring these hypotheses in this natural model of demyelinating disease. The significance of the difference in Caspr/paranodin-positive segment lengths in non-demyelinating regions between affected and control cattle brain remains obscure but alteration of this critical region is likely to have functional consequences on neurons.

### Pathological mechanisms resulting from *KIF1C* loss of function

Kinesin superfamily include more than 45 members [49], at least half of them being expressed in neuronal cells and most of them ubiquitously expressed. KIFs are major molecular motors of microtubule-based intracellular transport, and play a major role as stabilizers and depolymerizers of microtubules. These activities are important for cell division, cellular morphogenesis and mammalian development. Mutations in genes coding for several KIF proteins have already been associated with neurological diseases [50]. *KIF1C*, as a member of the kinesin-3 family which includes *Unc104/KIF1A*, *KIF1B*, *KIF14* and others [51], was initially reported to participate in transport of proteins from Golgi to endoplasmic reticulum [27], even if mice deficient for KIF1C exhibit no apparent cellular abnormality [14]. Recently, it was demonstrated that KIF1C transports Rab6 vesicles and can influence the Golgi complex structure, since its knock down slows the protein transfer to the cell surface [52].

The accumulation of membranous structures we observed in oligodendrocytes and in the centre of plaques in affected cattle could be a consequence of the perturbation of myelin membrane dynamics and/or trafficking. Similarly, a function of KIF1C in membrane dynamics, in association with microtubules, was demonstrated in podosome turnover in human macrophages [53,54] and vascular smooth muscle [55]. The absence of KIF1C led to podosome deficiency. Protein interaction studies have shown that KIF1C binds to non-muscle myosin IIA, providing an interface between the actin and tubulin cytoskeleton which facilitates podosome dynamics in a subcellular fine-tuned manner [53]. As the F-actin filament network is necessary for myelin wrapping of axons [56], it can be hypothesized that *KIF1C* loss of function alters membrane trafficking and membrane wrapping in oligodendrocytes along axons, as evidenced by the accumulation of actin in the plaques and in surrounding oligodendrocytes (S7 Fig).

In conclusion, we have identified the mutation causing progressive ataxia in cattle. This will be helpful for the Charolais breed in order to eradicate this disease, in France and worldwide, since this breed has been extensively exported. Loss of function of *KIF1C* is the source of a demyelination process that may explain the phenotype observed in animals that mimics spastic ataxia SPG58 in humans. As the mouse knock-out model does not develop a neurological phenotype, *KIF1C*-mutated cattle represent the unique natural model of SPG58 human pathology.

## Materials and methods

### Ethics statement

All studied animals originated from diverse Charolais herds throughout France, and were normally bred by the breeders. All samples and data were obtained with the oral consent of breeders or breed organizations.

Blood samples are those collected by veterinarians or by trained and licensed technicians during routine blood sampling for paternity testing, genomic selection or annual prophylaxis. All reported tissues were collected post-mortem after cattle were slaughtered for beef production at commercial slaughterhouses. No Institutional Animal Care and Use Committee (IACUC) or equivalent ethics committee(s) was required

### Bovine sample selection and DNA extraction

Seventy-one Charolais cattle presenting clinical signs compatible with progressive ataxia (i.e. unsteady gait, stiff hind limbs, irregular micturition in most of the female cases) were recorded by the French National Observatory of Genetic Diseases (S1 Table). Some of them were examined clinically by a veterinarian. When nervous tissues were available (i.e., for 10 of these 71 cases) histopathological analysis was done in order to confirm the disease diagnosis. Blood samples were collected from these cases and their parents, and DNA was extracted with a Genisol Maxi-Prep kit (ABgene, UK). Blood samples were also collected from their relatives and from unaffected animals. In total, 76 unaffected sires and dams were sampled.

### Homozygosity mapping

Forty-six affected animals were genotyped with the Bovine SNP50 Beadchip V1 (Illumina) at the Labogena facility. Mapping of the disease locus was carried out by homozygosity mapping with in-house software, as previously described [57].

### Whole-genome sequencing and analysis

Whole-genome sequencing was performed in 2012 at the Get-PlaGe platform (http://genomique.genotoul.fr/) on a HiSeq 2000 Illumina sequencer producing 100-bp-long paired-end reads, following the manufacturer’s protocol. Three animals had their genomic DNA sequenced: two sick animals aged respectively 31 and 36 months at last examination and one healthy animal (natural death at 10 years old). Reads were quality checked and mapped on the UMD3.1 reference genome using the BWA aln software (version 0.5.9-r16) [58]. The alignments were filtered with a minimum MAPQ value of 30. Reads that mapped to multiple localizations were removed. The target region identified by homozygosity mapping was selected on each produced bam file using Samtools (version 0.1.18) [59]. Local indel realignment and base quality recalibration were applied using GATK toolkit [60]. Single nucleotide polymorphisms (SNPs) were predicted with samtools mpileup and bcftools, and annotated with the Ensembl Variant Effect Predictor tool [61]. SNPs found in our studied breeds (Charolais, Limousin, Normande, Montbéliarde, Holstein, Blonde d’Aquitaine) were specified. Whole genome sequence data for the two sick animals are deposited in the European Nucleotide Archive under the study accession number: PRJEB27309.

### Sanger sequencing

To validate the variant identified after whole-genome sequencing in the *KIF1C* gene, the corresponding exon was first amplified using 200 ng of DNA, with standard GoTaq PCR reagents (Promega), on a Master Thermal Cycler (Eppendorf). Then, Sanger sequencing was performed according to standard protocols (Eurofins MWG). Primers, designed using Primer3, are available in S3 Table.

### Estimation of the allelic frequency of the *KIF1C* mutation

The *KIF1C* mutation identified in this study was included in duplicate in the Illumina EuroG10K custom SNP chip, which is routinely used for genomic selection in France. Thus, genotypes for this mutation were available for 61136 Holstein, 47048 Montbéliarde, 12891 Normande, 3597 Charolais, 2439 Blonde d’Aquitaine, 303 Limousin and 946 Brown Swiss animals.

### Association with growth and morphology traits

At the time of the analysis, Illumina EuroG10K SNP chip genotype data, including the ataxia test, were available for 3300 Charolais animals with growth and morphology data. The phenotypes included weight (kg) at birth, adjusted mean weight at 7, 18 and 24 months as well as muscular and skeletal development scores at 7 and 30 months (Table 3; S2 Table). Genotypes obtained with the Illumina EuroG10K SNP chip were available for approximately 3300, 3000, 450 and 250 individuals with phenotypes at birth, 7 months, 18 months and after two years of age, respectively. Phenotypes were corrected for environment effects (herd, year, season, dam parity) estimated in the national genetic evaluation system for beef cattle in the whole French Charolais population. Association between the ataxia variant and traits was then tested on these pre-corrected data with a mixed model including an overall mean, a random polygenic individual effect, the fixed effect of the genotype at the ataxia variant and a residual effect. The polygenic effect was estimated using a genetic relationship matrix built from pedigree data.

### Tissue collection

Affected calves were euthanized for ethical reasons by intravenous administration of euthanasia solution (T-61, embutramide 200 mg/mL, mebezonium iodure 50 mg/mL, tetracaine chlorhydrate 5 mg/mL, 1 dose of 0.1 mL/kg, Intervet, Angers, France), or in commercial slaughter houses by standard procedure. Tissue samples from the central nervous system (telencephalon, cerebellum and cervical spinal cord) were dissected and either frozen at -80°C for RNA/protein extraction, stored in RNALater (Quiagen) for RNA extraction, or fixed in formalin and then processed in a TP1020-1-1 tissue processor (Leica) and embedded in paraffin (EG 1160 Embedding Centre; Leica) or cryoprotected in 30% saccharose-PBS solution and frozen.

### RNA extraction–RT PCR

Total RNA was extracted from brain tissues using Qiazol reagent (Qiagen) and the RNeasy mini kit (Qiagen) with DNase I treatment. Then, 500 ng RNAs were reverse transcribed using an RT-vilo kit (Invitrogen), according to the manufacturer’s instructions.

To sequence transcripts, cDNA was amplified by PCR with PCR primers for *KIF1C* and ribosomal protein *RPL13* genes (S3 Table). Following PCR reactions, products were electrophoresed on a 1–2% agarose gel and amplified cDNA fragments of *KIF1C* were sequenced as indicated in a previous section.

### Immunoblot analysis

Bovine brain tissues were stored at -20°C in RNA-Later. Frozen tissues were grinded with plastic pestles in 1.5 mL Eppendorf tubes containing 50 mM Tris solution pH 7.4, SDS 2% and a protease inhibitor cocktail tablet (Roche Diagnostics). The protein extracts were centrifuged for 10 minutes at 16 000 g and 4°C. The supernatants were separated and protein contents were assayed with the 2-D Quant kit (GE Healthcare); 35–40 μg aliquots of proteins were mixed with 2 x Laemmli buffer, boiled for 5 min and frozen on ice. They were loaded on 10% SDS-PAGE and electrophoresed at 80 Volts for 20 min and 150 Volts for 50 min, with 10 μl of MW Marker (Nippon Genetics) added to the gel before electrophoresis. For western blot, proteins were transferred to a nitrocellulose membrane using a Trans-Blot Turbo apparatus (BioRad) for 10 min at 2.5 A and 25 Volts. The membrane was rinsed 2 x 5 min in MilliQ water and proteins were stained in 5x Ponceau Red solution for 5 min. Proteins were destained in TBS solution for 20 min and membrane sites were blocked in 5% dry milk TBS-0.1% Tween20 solution for 1 h at room temperature. The membrane was incubated with polyclonal rabbit antibody AKIN11 against KIF1C protein (Cytoskeleton Inc., Denver, CO) at a 1/500 ratio in TBS-0.1% Tween20 solution for 1 h at room temperature. After incubation, the membrane was washed 5 min and 2 x 10 min in fresh TBS-0.1% Tween20 solution. Then, the membrane was incubated with goat anti-rabbit horseradish peroxidase (HRP)-conjugated antibody (Santa Cruz) diluted at 1/20 000 in TBS-0.1% Tween20 solution for 1 h at room temperature (RT). The membrane was further washed as previously described. Bands were visualized by enhanced chemiluminescence (ECL Prime, GE Healthcare) and detected on ChemiDoc Touch (BioRad) in automatic mode.

Then, the membrane was washed 5 min in TBS solution, 2 x 10 min in TBS-0.1% Tween20 and was incubated with polyclonal antibody designed against ßactin, HRP conjugated (Sigma-Aldrich A3854) at a 1/30 000 ratio in TBS-0.1% Tween20 solution for 1 h at room temperature. The following steps were as previously described.

### Histochemistry and immunohistochemistry

A full neuropathological examination was conducted according to classical methods, using frozen and paraffin-embedded sections of brain structures. Samples from motor cortex, internal capsules, cerebellum, pons and cervical spinal cord from affected and heathy cattle were obtained.

Frozen sections were stained for neutral fat with Sudan black and Sudan III. Paraffin sections were stained for cytology analysis using Nissl or hemalun-eosin-safran (HES) staining. HES staining was carried out in a VV24/4 VARISTAIN automatic slide stainer (Thermo Electron) according to a standard protocol. Klüver–Barrera staining for myelin and Congo red and thioflavin staining for amyloid fibrils were also performed, using specific protocols. After staining, slides were scanned on a 3DHISTECH scanner (Sysmex).

Immunostaining was performed both on paraffin-embedded and frozen sections using the citrate buffer epitope retrieval method. Endogenous peroxidase was quenched by incubation for 20 min at RT in a PBS/0.1% Triton X-100 (Sigma) solution containing 10% methanol and 0.003% H2O2. Microscope slides carrying brain sections were washed three times and incubated in the blocking solution (0.1% PBS, 4%Triton X-100, 4% normal goat serum [NGS], 2% bovine serum albumin [BSA]) for 1 h at RT. Sections were then incubated for 48 h at 4°C with specific antibodies (S4 Table) diluted in blocking solution against the following proteins: calcium binding protein 28 Kd (calbindin 28 Kd, a marker of Purkinje cells), glial fibrillary acidic protein (GFAp, an astrocytic marker), calcium binding protein 17 Kd (Iba-1, a marker of macrophages/microglia), myelin basic protein (MBP, a major component of myelin sheaths), oligodendrocyte-specific protein (OSP, a component of oligodendrocyte plasma membrane), cluster of differentiation 3ε (CD3, a marker of T lymphocytes), against Caspr/paranodin (an axonal component and marker of the paranodal axoglial junction) and anti-actin. The sections were then incubated for 2h at RT with the corresponding biotinylated appropriate (anti-mouse or anti-rabbit) secondary antibody (1:250; Vector Laboratories) diluted in blocking solution. Bound antibodies were alternatively visualized using the ABC amplification system (Vectastain ABC kit, Vector Laboratories) with 3,3'-diaminobenzidine tetrahydrochloride (DAB Metal Concentrate; BioGenex) as the substrate, or by fluorochrome FITC or Cy3. The sections were alternatively counterstained with haematoxylin or DAPI. They were mounted directly in Moviol when fluorochrome was used or dehydrated in ethanol and xylene solutions and mounted with Eukitt when DAB chromogen was used.

Neuronal loss, gliosis and atrophy were semi-quantitatively assessed and categorized by two different observers as severe, moderate or absent.

### Morphometry of Caspr/paranodin aggregates

The length of immunolabelling of Caspr/paranodin aggregates on cryostat or paraffin sections was measured in the cerebellum and internal capsule. The measurements were performed on images obtained by NanoZoomer 2.O-RS (Hamamatsu) apotome equipped with 20X and 40X objectives or a Leica DM400B optical microscope equipped with 40X, 63X and 100X objectives, according to the chromogen used, respectively fluoroscein, CY3 or DAB. Images acquired from epifluorescence or light microscope were superimposed using ImageJ. The length of paranodin immunostaining was measured inside and outside the plaque in affected and control tissues; the lengths were compared using a variance analysis with Anova.

### Electron microscopy

Brain samples from affected case #55 including the cerebellum, internal capsule and spinal cord were further processed for 24-h fixation in 2% paraformaldehyde and 1% glutaraldehyde for conventional electron microscopy study. After post-fixation in 1% osmium tetroxide, blocks were embedded in Araldite. Semi-thin sections about 0.5 μm thick were stained with toluidine blue. Ultrathin sections were counterstained with uranyl acetate and lead citrate and examined under a Hitachi HT 7700 electron microscope (Elexience, Verrières-le-Buisson, France).

## Supporting information

S1 Fig Pedigree presenting a proband case born in 2005 representative ataxic case (coloured in grey) and its relationships with the predominant founder ancestor, born in 1964. Most of the affected cases could be traced back to this ancestor, but for an easier representation, the pedigree presents only one case.
(TIF)

S2 Fig Homozygosity mapping results leading to the identification of a 681-kb homozygous interval of chromosome 19 as the locus containing the mutation of progressive ataxia in the Charolais breed. The peak of BTA17 corresponds to a single marker above the threshold, without a homozygous interval around it.
(TIF)

S3 Fig *KIF1C* exons 5 and 6 were analysed by ESE finder software. The analysis suggests that the mutation affects ESE (exonic splicing enhancer) motifs and may modify the affinity for different SR proteins (SRSF1, SRFS2, SRSF6). Binding scores for different splicing proteins are shown on the y-axis; Nucleotides are shown on the x-axis.
(TIF)

S4 Fig Sequences of KIF1C wild type protein, and of the two types of proteins resulting from *KIF1C* mutation. (A) Sequence of bovine KIF1C wild type protein. (B) The mutated protein with one amino acid substitution. (C) The truncated protein resulting from exon 5 splicing and premature stop codon.
(TIF)

S5 Fig Histological characteristics of lesion. (A) Kluver-Barrera (KB) staining of frontal section of cerebellar folia; WML, white matter; GCL, granule cell; PCL, Purkinje cells; ML, molecular layers. White matter in longitudinal section strewn with plaques of myelin pallor (*red asterisks*). The demyelinating plaques were often confluent (*lined up red asterisks*). The shape of the lesions is often ovoid and their size variable from 10 to 80 μm in diameter. These lesions were seen in all studied myelin tracts (cerebellum, corpus callosum, internal capsule, spinal cord and bulbar tracts). (B) Several plaques of various shapes and diameters in cerebellar white matter. Plaques are slightly eosinophilic; paraffin-embedded section and Hemalun-Eosin-Safran staining. (C) Cervical spinal cord on frontal section KB stained, some neuronal fibres seem to by-pass the myelin pallor plaques, the centre of some of which contains material stained by the lipophilic KB talc. (D) Sudan black staining of longitudinal section of demyelinating plaque in internal capsule showing lipidic granules and cellular debris in the centre (*arrows*) and also staining of axons on the periphery and crossing stack (*arrow head*). (E) Neural fibres of cerebellar white matter stained by calbindin antibody showed that stacks are by-passed by some of them (*arrow*). However, some nervous fibres could pass through the stack. Scale bar: (A) 200 μm, (B) and (C) 100 μm, (D) 30 μm, (E) 80 μm.
(TIF)

S6 Fig Mutations of *KIF1C* gene in human and bovine. **Adapted from** [11]. Mutations of human *KIF1C* resulting in hereditary spastic paraplegia cases are presented: mutations published by [11] are written in grey, mutations published by [32] are written in blue, mutations published by [12]are written in green, mutations published by [13] are written in black. Mutation of bovine *KIF1C* resulting in progressive ataxia is presented in red.
(TIF)

S7 Fig Hypothesis of pathological mechanism in bovine progressive ataxia cases, resulting from the *KIF1C* mutation. Oligodendrocytes wrap their plasma membrane around axons and then generate multi-lamellar sheaths of myelin. The leading edge protrusion of oligodendrocytes and its repetitive rotational movement depends on the strength of F-actin turnover. This mechanism may explain early myelination processes. The molecular mechanism driving the maintenance of myelination in the adult is not well known but a similar mechanism could operate. In *KIF1C*-mutated Charolais cattle the absence of KIF1C, which transports G-actin along microtubules towards peripheral processes and the leading edge, the cellular distribution of actin was perturbed in oligodendrocytes, and the sick cell could not maintain the myelin sheaths surrounding axons.
(TIF)

S1 Table Characteristics of the 71 Charolais cattle suspected of progressive ataxia and five control animals used in this study. (na: not available).
(XLSX)

S2 Table Association between the ataxia variant and morphology traits in animals of the Charolais breed. n(GG) stands for the number of animals homozygous for the WT (G) allele; n(AA) for the number of animals homozygous for the ataxia (A) allele; n(AG) for the number of heterozygous animals. AA-GG: contrast between ataxia (AA) and WT (GG) genotypes; AA-GG mean and AA-GG std: effect of the ataxia allele (at the homozygous state) respectively on the phenotypic mean and standard deviation.
(XLSX)

S3 Table Primers used for Sanger sequencing and RT-PCR. (XLSX)

S4 Table Primary antibodies and histochemistry staining used in the analysis on bovine tissues. Many antibodies do not reveal antigenicity in bovine samples, probably due to non-cross-reactivity between the species.
(XLSX)
