# Supplementary figures and images for: Progressive ataxia of Charolais cattle highlights a role of KIF1C in sustainable myelination
Source: PLoS Genet. 2018 Aug 1;14(8):e1007550. doi: 10.1371/journal.pgen.1007550 (PMC6089448; doi:10.1371/journal.pgen.1007550)

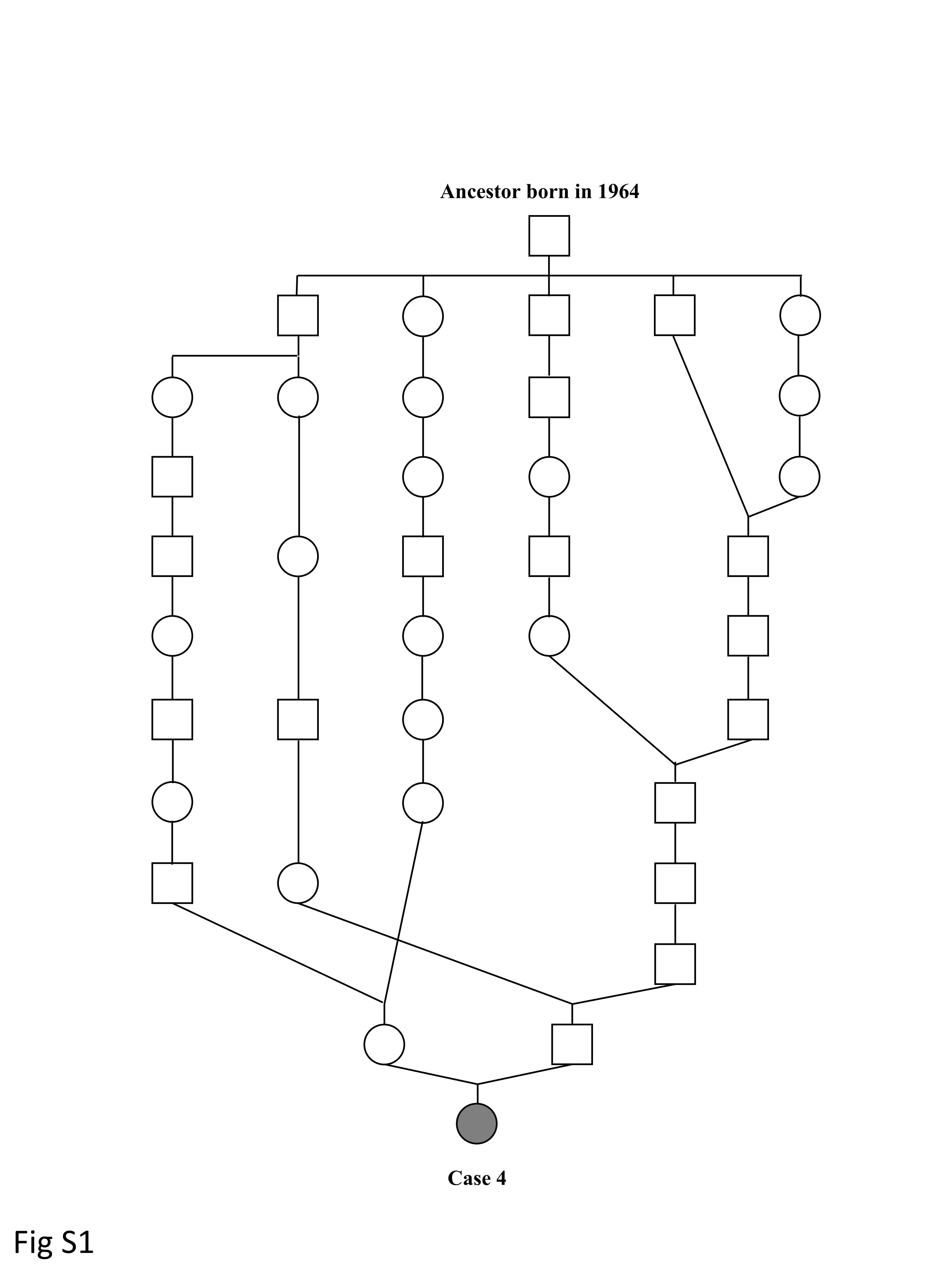

Supplement: S1 Fig — Most of the affected cases could be traced back to this ancestor, but for an easier representation, the pedigree presents only one case. (TIF) [file pgen.1007550.s001.tif]

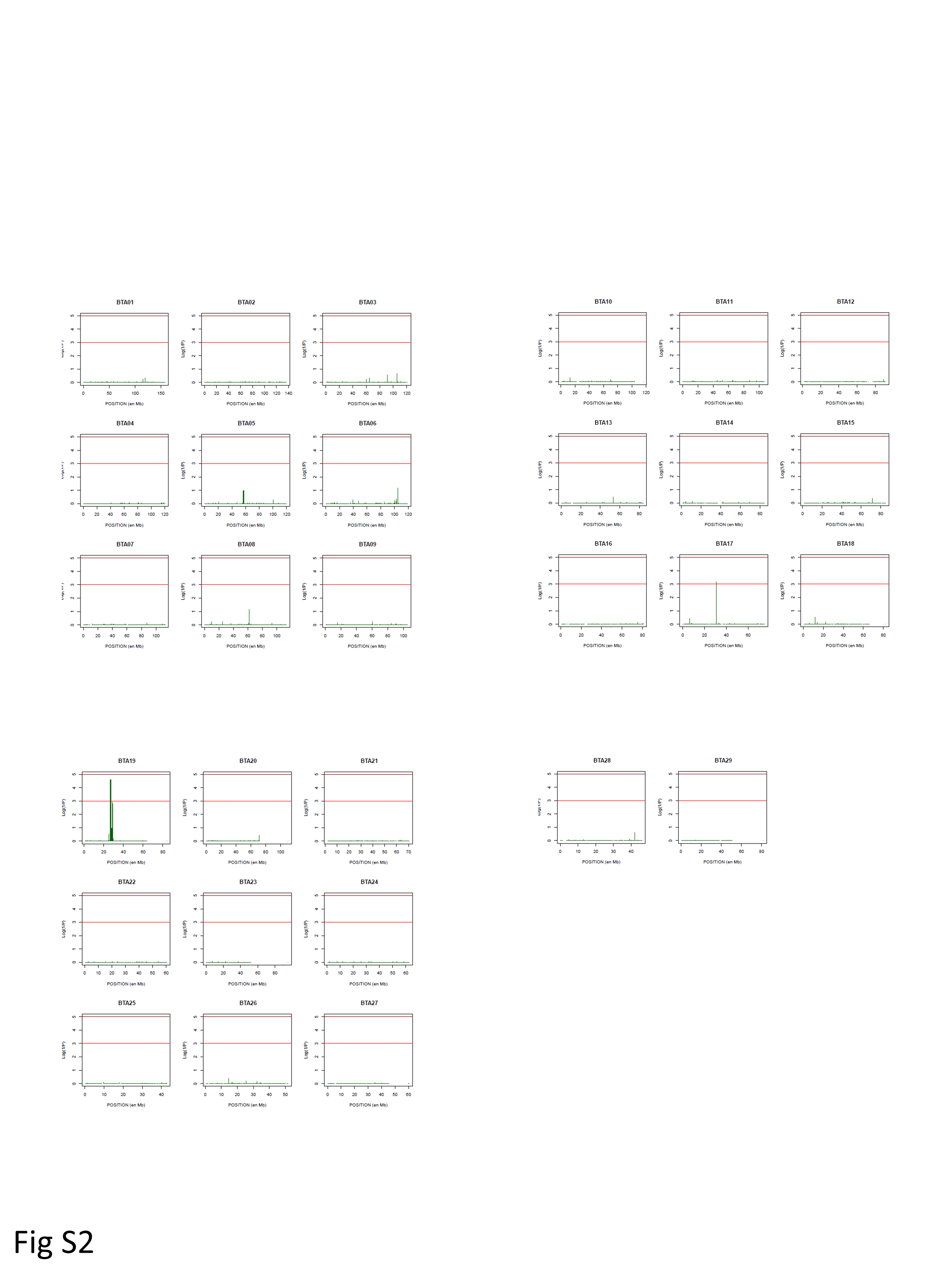

Supplement: S2 Fig — The peak of BTA17 corresponds to a single marker above the threshold, without a homozygous interval around it. (TIF) [file pgen.1007550.s002.tif]

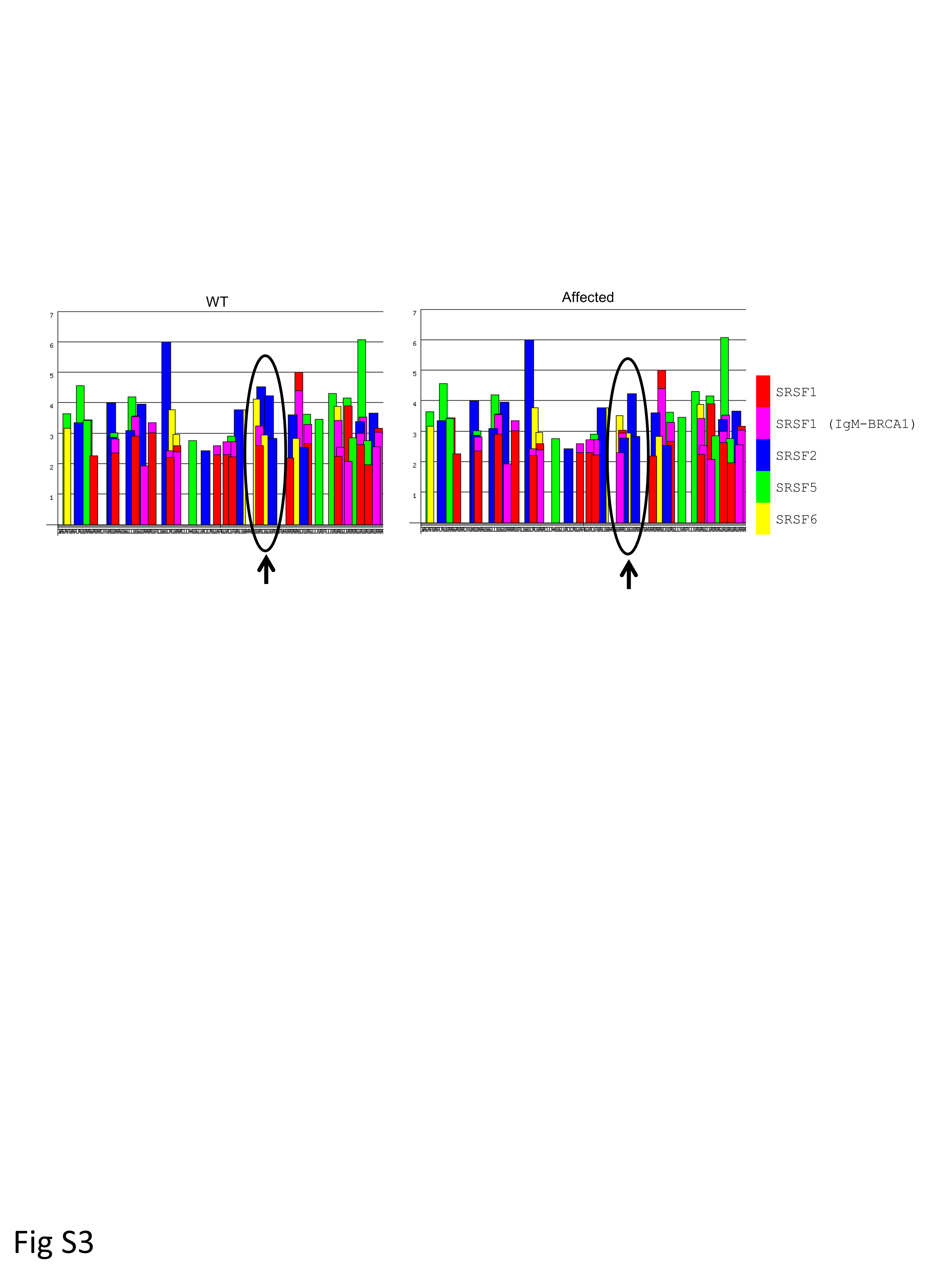

Supplement: S3 Fig — The analysis suggests that the mutation affects ESE (exonic splicing enhancer) motifs and may modify the affinity for different SR proteins (SRSF1, SRFS2, SRSF6). Binding scores for different splicing proteins are shown on the y-axis; Nucleotides are shown on the x-axis. (TIF) [file pgen.1007550.s003.tif]

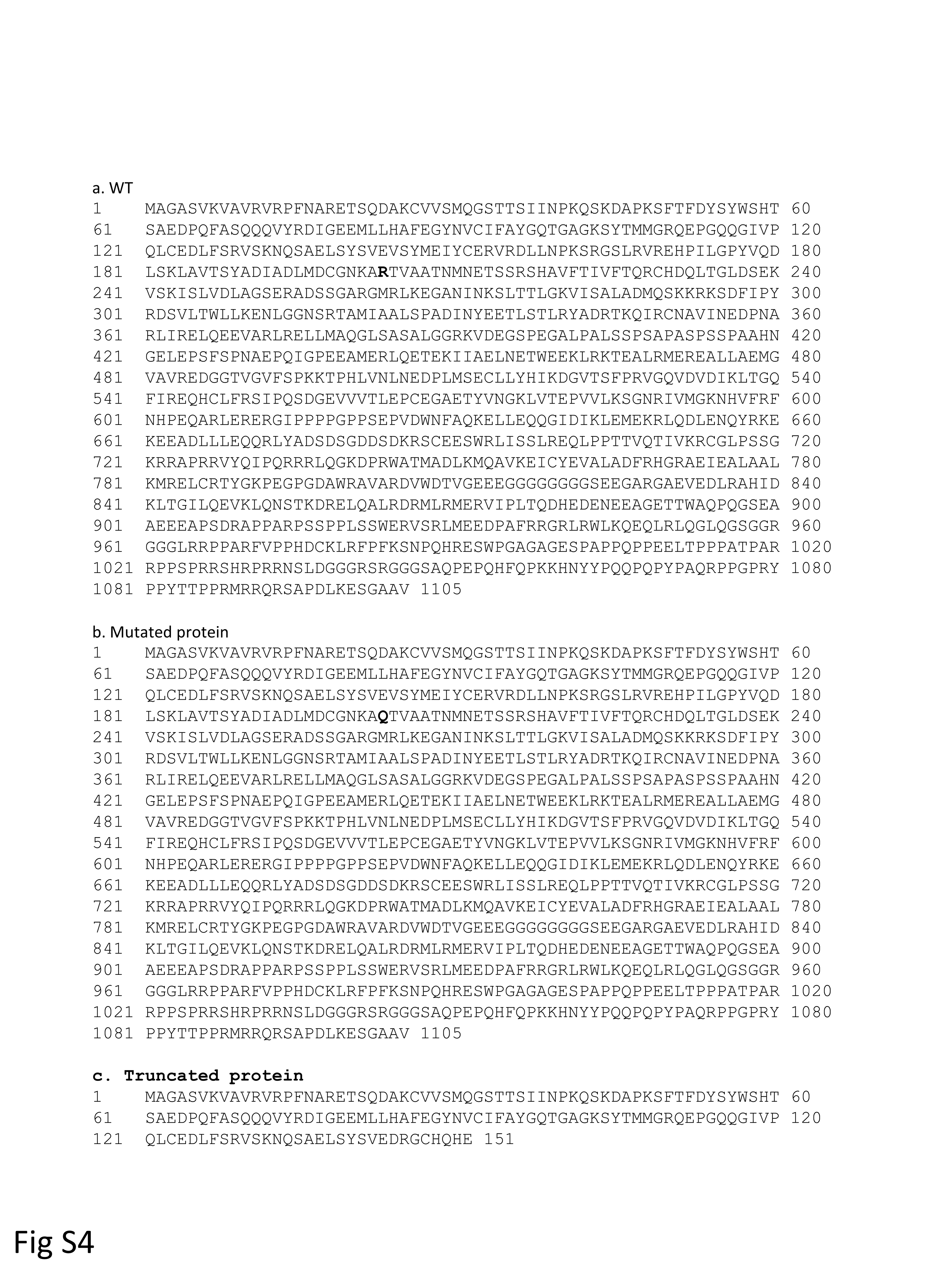

Supplement: S4 Fig — (A) Sequence of bovine KIF1C wild type protein. (B) The mutated protein with one amino acid substitution. (C) The truncated protein resulting from exon 5 splicing and premature stop codon. (TIF) [file pgen.1007550.s004.tif]

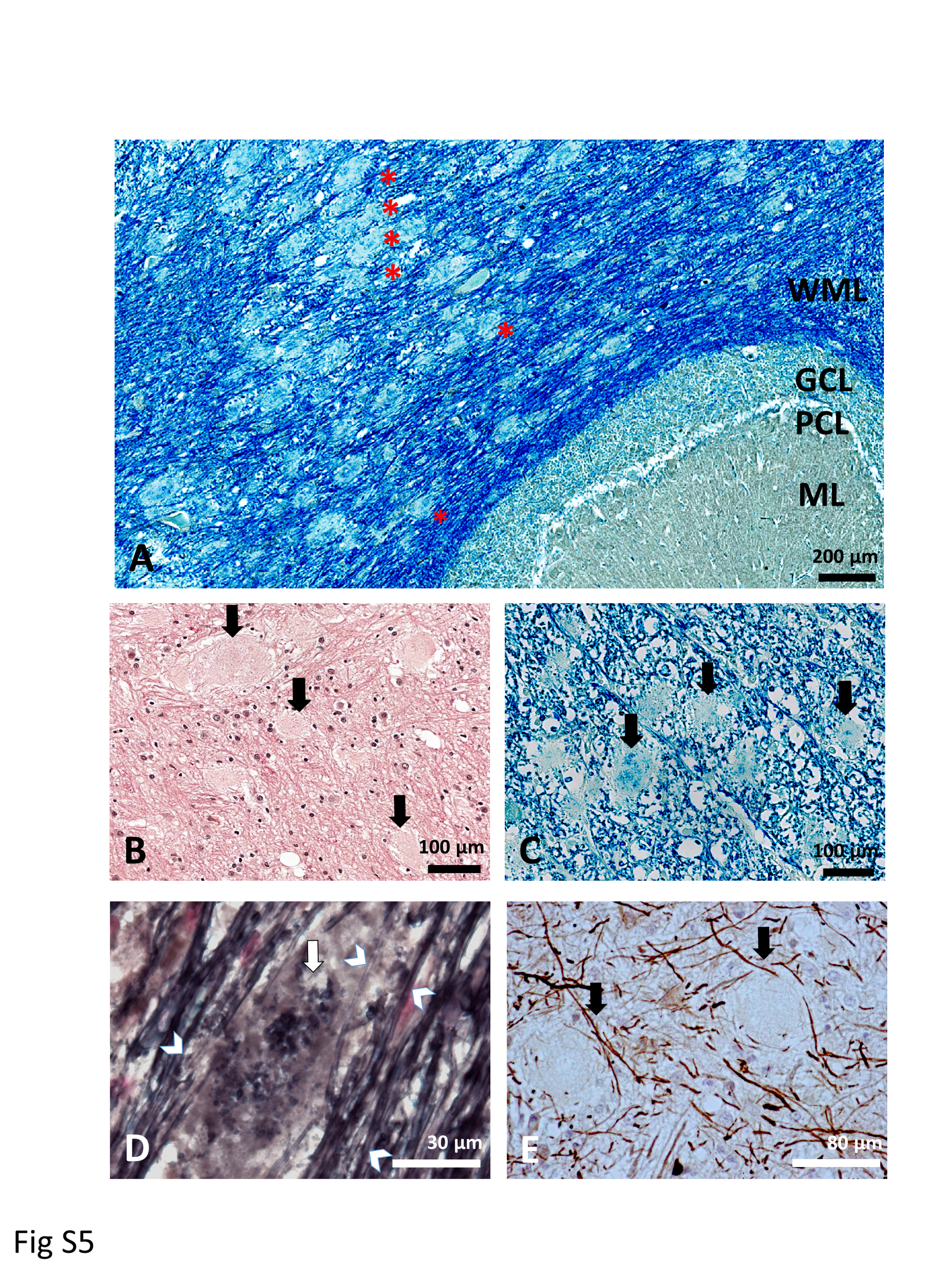

Supplement: S5 Fig — (A) Kluver-Barrera (KB) staining of frontal section of cerebellar folia; WML, white matter; GCL, granule cell; PCL, Purkinje cells; ML, molecular layers. White matter in longitudinal section strewn with plaques of myelin pallor (red asterisks). The demyelinating plaques were often confluent (lined up red asterisks). The shape of the lesions is often ovoid and their size variable from 10 to 80 μm in diameter. These lesions were seen in all studied myelin tracts (cerebellum, corpus callosum, internal capsule, spinal cord and bulbar tracts). (B) Several plaques of various shapes and diameters in cerebellar white matter. Plaques are slightly eosinophilic; paraffin-embedded section and Hemalun-Eosin-Safran staining. (C) Cervical spinal cord on frontal section KB stained, some neuronal fibres seem to by-pass the myelin pallor plaques, the centre of some of which contains material stained by the lipophilic KB talc. (D) Sudan black staining of longitudinal section of demyelinating plaque in internal capsule showing lipidic granules and cellular debris in the centre (arrows) and also staining of axons on the periphery and crossing stack (arrow head). (E) Neural fibres of cerebellar white matter stained by calbindin antibody showed that stacks are by-passed by some of them (arrow). However, some nervous fibres could pass through the stack. Scale bar: (A) 200 μm, (B) and (C) 100 μm, (D) 30 μm, (E) 80 μm. (TIF) [file pgen.1007550.s005.tif]

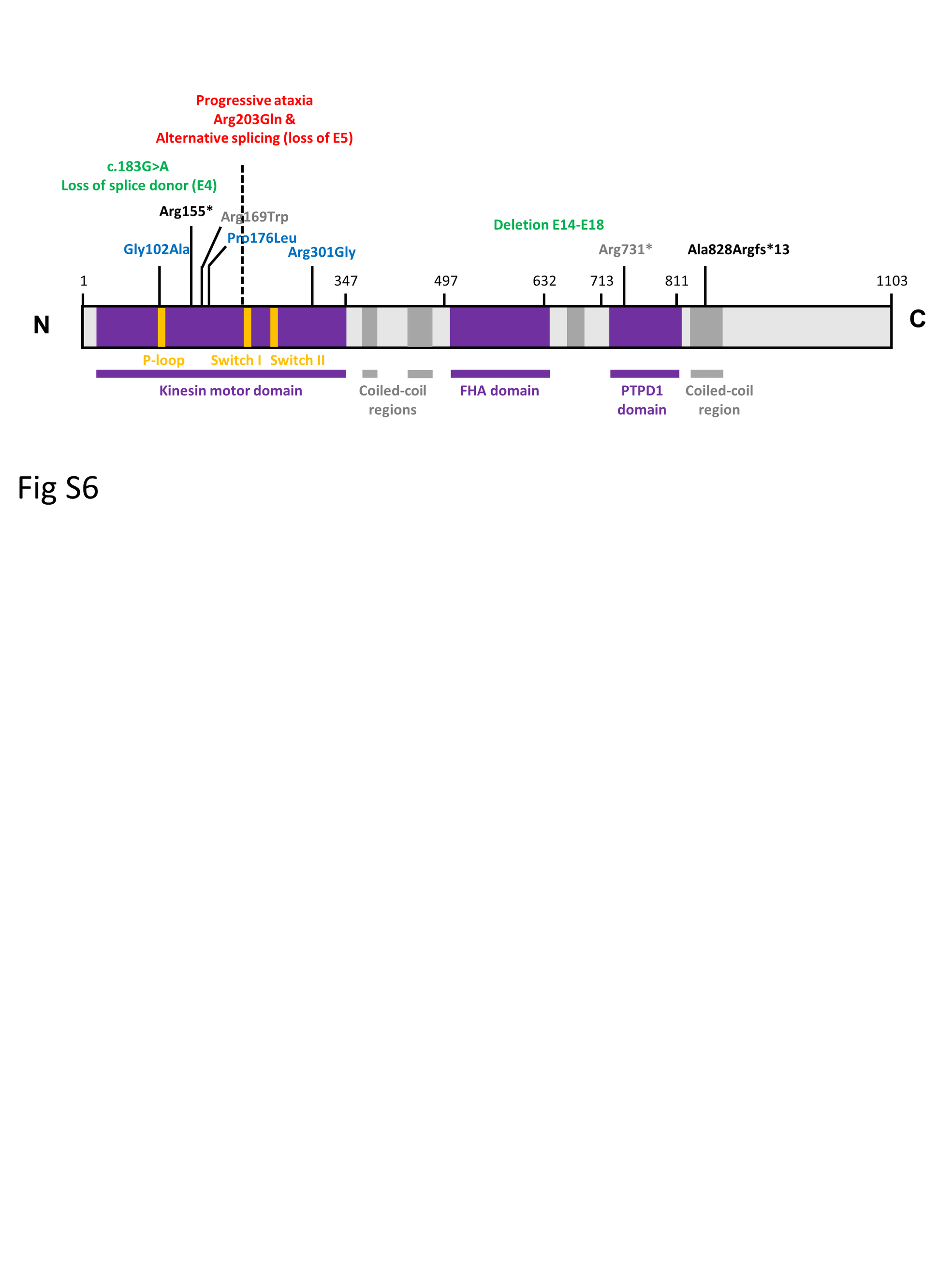

Supplement: S6 Fig — Adapted from [11]. Mutations of human KIF1C resulting in hereditary spastic paraplegia cases are presented: mutations published by [11] are written in grey, mutations published by [32] are written in blue, mutations published by [12]are written in green, mutations published by [13] are written in black. Mutation of bovine KIF1C resulting in progressive ataxia is presented in red. (TIF) [file pgen.1007550.s006.tif]

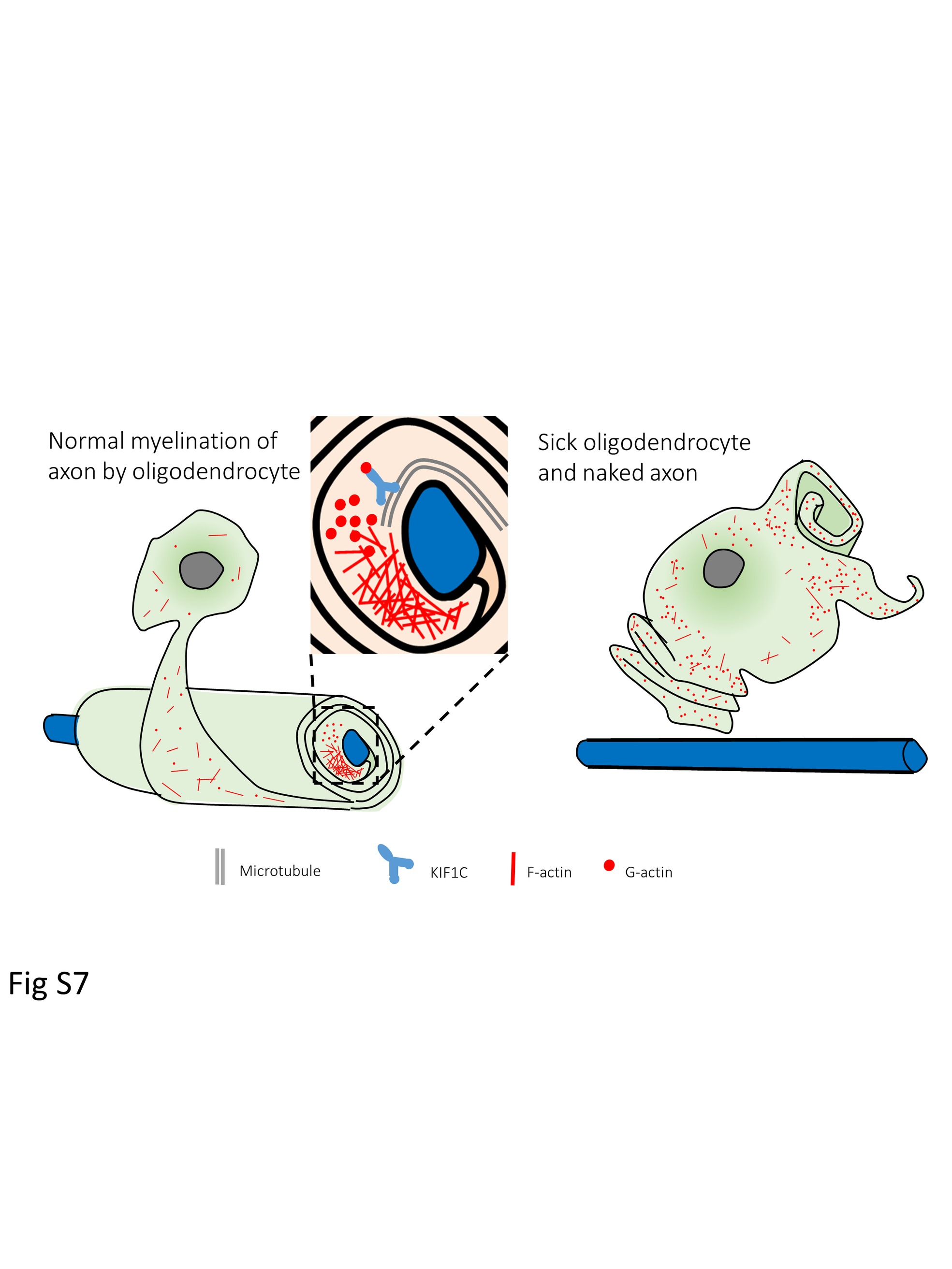

Supplement: S7 Fig — Oligodendrocytes wrap their plasma membrane around axons and then generate multi-lamellar sheaths of myelin. The leading edge protrusion of oligodendrocytes and its repetitive rotational movement depends on the strength of F-actin turnover. This mechanism may explain early myelination processes. The molecular mechanism driving the maintenance of myelination in the adult is not well known but a similar mechanism could operate. In KIF1C-mutated Charolais cattle the absence of KIF1C, which transports G-actin along microtubules towards peripheral processes and the leading edge, the cellular distribution of actin was perturbed in oligodendrocytes, and the sick cell could not maintain the myelin sheaths surrounding axons. (TIF) [file pgen.1007550.s007.tif]
